# A *Semi*-In Vivo System Reveals Impairment of Cell-Cycle Machinery upon Derailment of Polarity and Cellular Differentiation in In Vitro-Fertilised Egg Cells of Wheat (*Triticum aestivum* L.)

**DOI:** 10.3390/ijms27219612

**Published:** 2026-10-27

**Authors:** Zsolt Pónya, Iman Mirmazloum, Sándor Keszthelyi, Sándor Farkas, Richárd Hoffmann, György Bázár, Mauro Cresti

**Affiliations:** 1 Institute of Agronomy, Hungarian University of Agriculture and Life Sciences (MATE), 40 Guba Sándor Street, 7400 Kaposvár, Hungary; keszthelyi.sandor@uni-mate.hu (S.K.);; 2 Department of Plant Physiology and Plant Ecology, Institute of Agronomy, Hungarian University of Agriculture and Life Sciences (MATE), 44 Ménesi road, 1118 Budapest, Hungary; 3 Department of Nature Conservation Biology, Institute for Wildlife Management and Nature Conservation, Hungarian University of Agriculture and Life Sciences (MATE), Kaposvár Campus, 40 Guba Sándor Street, 7400 Kaposvár, Hungary; 4 Hungarian University of Agriculture and Life Sciences (MATE) Innovation Kft., 1 Páter Károly Street, 2100 Gödöllő, Hungary; 5 Department of Life Sciences, University of Siena, Via Mattioli 4, 53100 Siena, Italy

**Keywords:** egg cell polarity, CDC2 kinase, cell-cycle, in vitro fertilisation, microinjection, wheat zygotic embryogenesis

## Abstract

Cyclin-dependent kinases (CDKs) are central regulators of cell-cycle progression and play essential roles in coordinating DNA replication, mitosis and cytokinesis. Although their involvement in cell proliferation has been extensively investigated, considerably less is known about their contribution to developmental pattern formation during the earliest stages of plant embryogenesis. This question is particularly relevant in angiosperms, where the first zygotic division is intrinsically linked to cell differentiation and formation of the apical–basal body axis. In the present study, the localisation and functional significance of the wheat CDC2 (Cell Division Cycle 2) homologue, p34cdc2, the key regulator of the G2/M transition, were investigated in in vivo- and in vitro-fertilised egg cells of wheat (*Triticum aestivum* L.). An affinity-purified antibody raised against a synthetic peptide corresponding to the C-terminal region of wheat CDC2 enabled the immunolocalisation of p34cdc2 during zygote development and early embryogenesis. Double-labelling experiments with tubulin showed stage-associated differences in p34cdc2 localisation and its apparent association with the preprophase band, a cytoskeletal structure involved in division-plane determination. Functional analyses showed that pharmacological and antibody-based perturbations were associated with marked changes in early embryonic development. Microinjection of anti-CDC2 and anti-PSTAIRE antibodies into in vitro-fertilised egg cells revealed a critical developmental window shortly after fertilisation during which antibody-mediated interference markedly reduced subsequent cell division. Likewise, inhibition of CDKs by purine derivatives, including olomoucine, disrupted normal morphogenesis and prevented establishment of characteristic embryonic organisation. Oryzalin-mediated disruption of microtubules was accompanied by loss of detectable p34cdc2 localisation to the future division plane, whereas genistein treatment induced severe polarity defects and aberrant embryonic pattern formation. An elaborate semi-in vivo re-implantation system indicated that maternal ovular tissues can support the maintenance of polarity and embryogenic competence during zygote and proembryo development. Taken together, our findings indicate that CDC2-associated processes are closely linked to cell-cycle progression and may be coordinated with polarity establishment and embryonic pattern formation. These results support a model in which interference with CDC2-associated processes can be accompanied by disrupted cell-cycle progression and abnormal embryonic patterning during early wheat embryogenesis.

## 1. Introduction

Cell division is one of the most substantial biological processes, and its regulation displays remarkable evolutionary conservation across the entire spectrum of living systems [1]. The coordinated interaction of multiple regulatory networks that synchronise DNA replication, transcription, chromosome segregation and cytokinesis is a prerequisite for proper cell-cycle progression [2,3,4,5]. Cell division and cellular differentiation are often considered as two separate events, but they co-occur during zygote formation in angiosperms; nonetheless, their relationship during the earliest stages of development remains incompletely understood [6,7].

This issue is of particular importance in early embryogenesis, especially in higher plants in which the first cell division of the zygote is coupled to cell differentiation as the zygotic cleavage yields two daughter cells of distinct cell fates. In flowering plants, the first zygotic division represents both a mitotic event and the earliest visible manifestation of developmental pattern formation. This asymmetrical division is a prerequisite for formation of the apical–basal axis of the angiosperm proembryo. This elaborate synchronisation can only be achieved if morphogenesis checkpoints impact cell-cycle progression to attain the required developmental pattern coupled to successful proliferation. In a developmental context, when analysing key regulators of the cell cycle, the delicate balance among the cell division cycle, differentiation and morphogenesis must be considered.

Increasing evidence points to the possibility that the molecular and structural asymmetries underlying this process are already present in the egg cell and are maintained in the zygote prior to cell cleavage, indicating that intracellular polarisation precedes embryonic axis formation [8,9,10,11,12,13]. More recent molecular studies have identified several regulatory pathways involved in specifying zygotic polarity. In *Arabidopsis* [14,15,16,17,18,19,20], these include signalling through YODA (YDA), a mitogen-activated protein kinase kinase kinase (MAPKKK) involved in basal lineage/extra-embryonic fate, and downstream mitogen-activated protein kinases (MAPKs), together with members of the WUSCHEL-RELATED HOMEOBOX (WOX) family of transcription factors and receptor kinase-mediated signalling pathways [21]. These pathways contribute to zygote elongation, asymmetric division and the subsequent specification of apical and basal cell fates. Cytoskeletal organisation also contributes directly to the spatial control of the first zygotic division. For instance, in tobacco, the dynamin-related protein NtDRP was shown to be required for correct orientation of the zygotic division plane and subsequent differentiation of the basal cell lineage, apparently through its effects on microtubule organisation and spindle orientation. When analysing the role of cell-cycle regulators during development, the relationship between cell proliferation, differentiation and morphogenesis must be considered [22,23,24]. Thus, studies aimed at attempting to disentangle the complicated molecular and cellular mechanisms rendering a cell capable of progressing from simple to more complex, differentiated forms can be assisted by experimental approaches based on the alteration of cell-cycle progression [22,25,26,27,28,29,30,31,32,33]. Successful development requires continuous coordination between cell-cycle progression and morphogenetic processes, implying the existence of checkpoint mechanisms capable of integrating spatial information with proliferation control. Although extensive research has focused on DNA replication checkpoints and genome integrity surveillance, considerably less is known about how morphogenetic processes influence cell-cycle progression during embryogenesis.

Evidence for such interactions has emerged from yeast and other eukaryotic systems. In budding yeast, morphogenesis checkpoints coordinate cytoskeletal organisation with cell-cycle progression [3,13,22,25,26,27,28,29,30,31,32,34]. More generally, cell-cycle regulators are closely integrated with developmental and differentiation programmes [14,15,16,17,18,19,20,35,36,37,38,39]. In plants, however, our understanding of these relationships, including potential links between cell-wall integrity and cell-cycle progression, remains comparatively limited, despite increasing evidence that cell division, polarity and differentiation must be tightly coordinated during embryogenesis.

One major reason these mechanisms remain poorly understood is the inaccessibility of the angiosperm female gamete and zygote. Unlike in many animal systems, fertilisation and early embryogenesis in angiosperms occur deeply embedded within maternal tissues, making direct observation and experimental manipulation difficult [14,15,16,17,18,19,20,35,36,37,40,41,42,43]. Consequently, the molecular mechanisms coupling cell-cycle progression to polarity formation and cellular differentiation during the earliest stages of plant embryogenesis remain incompletely understood.

A central component of eukaryotic cell-cycle control is the highly conserved cyclin-dependent kinase CDC2, a key regulator of cell-cycle transitions [31,32,33,44,45,46,47,48,49]. CDC2-related kinases act through reversible phosphorylation of substrates involved in DNA replication, chromosome condensation, spindle formation and cytokinesis [22,23,24,50,51,52,53,54]. Through interactions with cyclins and associated regulatory proteins, CDKs coordinate progression through the major phases of the cell cycle. Increasing evidence also links CDK activity to developmental processes extending beyond classical cell-cycle regulation, including differentiation and cellular pattern formation.

Particularly intriguing is the possibility that CDC2-associated processes contribute not only to mitotic progression but also to developmental pattern formation during embryogenesis. In plants, the first division of the zygote is unique because it simultaneously represents a cell-cycle event and the first visible expression of developmental patterning in the sporophytic generation. Following fertilisation, the zygote undergoes polarisation and elongation before executing an asymmetric division that produces the apical and basal daughter cells. Increasing evidence suggests that the molecular and structural asymmetries underlying apical–basal axis formation are already established in the egg cell and are maintained upon sperm–egg cell fusion throughout zygote formation and cell division. This reinforced polarity is reflected in the first asymmetric division of the zygote [14,15,16,17,18,19,20,35,36,37], which initiates lineage specification, ultimately giving rise to the embryo proper and suspensor. Consequently, perturbation of the molecular machinery controlling this developmental transition may lead not only to the derailment of cell proliferation but also to that of embryonic pattern formation.

The complexity of this problem is further compounded by the fact that embryonic cell cycles can differ substantially from those operating in somatic tissues, including differences in checkpoint control and in the regulation of CDK activity [33,46,55,56,57,58,59,60,61]. In the brown alga Fucus, the first zygotic cell cycle displays distinctive translational regulation of PSTAIRE CDKs, and inhibition of CDK-like proteins with purine derivatives or anti-PSTAIRE antibody interferes with cell-cycle progression and polarised development. These observations indicate that CDK-associated processes can be closely coordinated with early developmental patterning and provide a useful comparative framework for investigating the corresponding relationship in angiosperm zygotes.

Although considerable advances have been made in our understanding of embryo patterning in Arabidopsis, relatively little is known about the molecular mechanisms regulating polarity development and cell-cycle progression in cereal species. Wheat embryogenesis takes place within a highly specialised maternal environment, and the orientation and polarity of the egg cell and zygote are closely associated with formation of the embryonic apical–basal axis [15,17,18,19,20,35,36,37]. Maternal and extra-embryonic signalling also contributes to early embryo patterning in flowering plants [40,41,42,43,62].

A major obstacle to the experimental investigation of these questions has been the limited accessibility of the angiosperm egg cell and zygote. The development of in vitro fertilisation systems represented a significant advance because it enabled direct manipulation of isolated gametes and controlled generation of zygotes under experimental conditions. Isolated wheat zygotes can also be monitored individually throughout early development, providing access to cellular events occurring immediately after fertilisation. However, removal from the native ovular environment also eliminates potential positional and maternal cues, which must be considered when interpreting polarity and pattern formation in isolated culture.

Nevertheless, what putative signals derive from the maternal tissue during egg cell activation/zygote development in the course of zygotic cleavage/proembryo formation in angiosperms still awaits elucidation. In order to maintain the apical–basal polarity in the angiosperm egg/zygote that is oriented relative to maternal structures, thereby ensuring that cues stemming from the ovular tissues (believed to play an important role in the orientation of developmental signals during the early steps of zygotic embryogenesis) are present during zygotic development of in vitro-fertilised wheat egg cells, we employed a “re-implantation” technique elaborated in our laboratory that allowed for in situ development of the in vitro-fused gamete pairs, hence facilitating the contact of the developing zygote with the maternal tissue.

Using this system in combination with immunolocalisation, confocal microscopy, pharmacological inhibition and antibody microinjection, we investigated the localisation and functional significance of the wheat CDC2 homologue, p34^cdc2^, during fertilisation and early embryogenesis. Specifically, we sought to determine whether processes associated with CDC2 activity participate exclusively in cell-cycle progression or whether they also contribute to developmental processes associated with polarity development, asymmetric zygotic division and embryonic pattern formation. By combining localisation studies with functional perturbation experiments, we aimed to test the hypothesis that disruption of CDC2 activity interferes with the formation and stabilisation of developmental polarity, thereby uncoupling normal embryonic pattern formation from cell-cycle progression during early wheat embryogenesis.

## 2. Results

### 2.1. Validation of the Affinity-Purified Antibody Against Wheat p34cdc2

**Figure 1:**
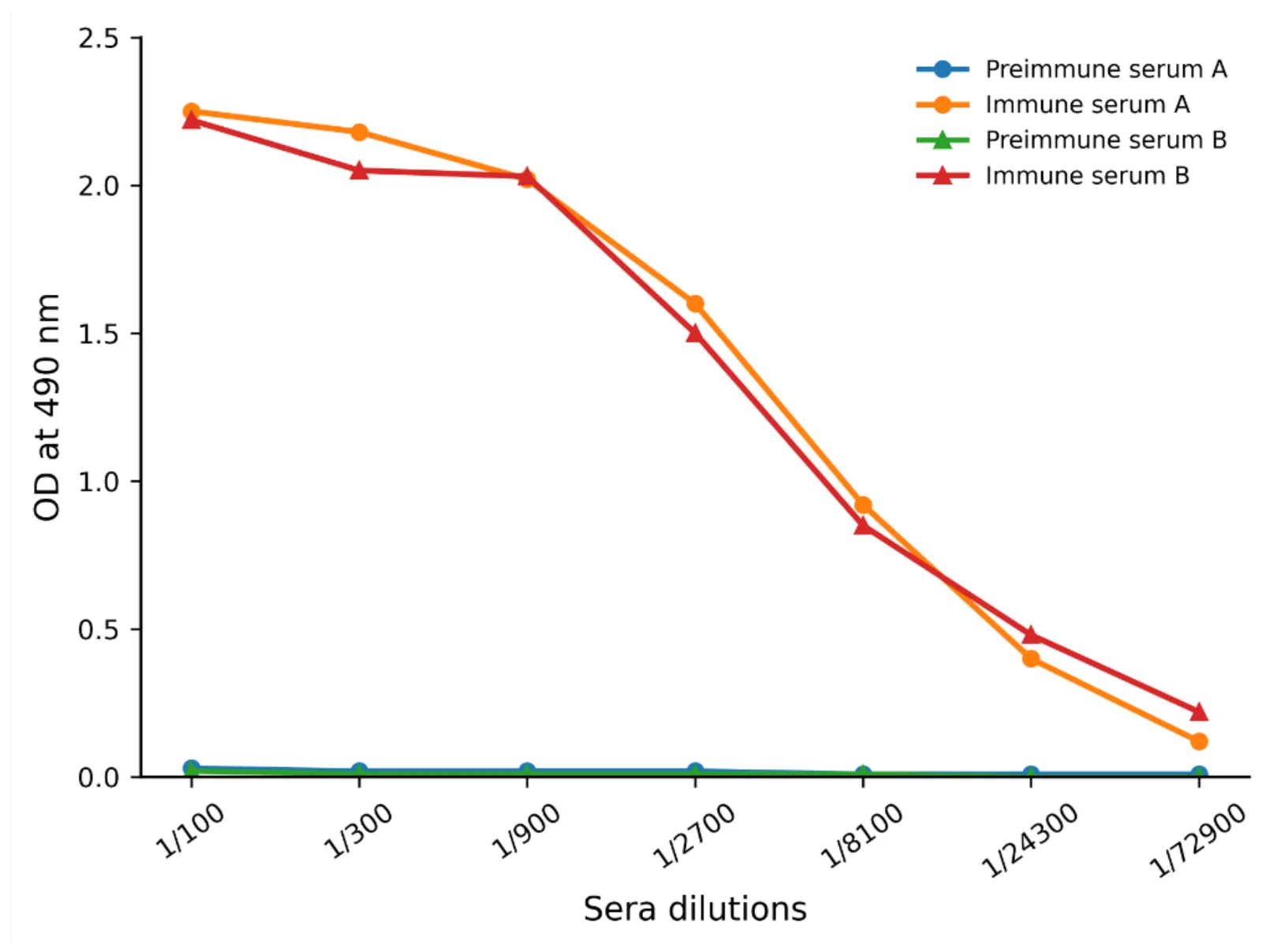
ELISA test results of the antibody produced in a rabbit against the 17-C terminal peptide deduced from the wheat CDC2 protein.

To investigate the possible involvement of CDC2-associated processes during fertilisation and early embryogenesis in wheat, an antibody was raised against a synthetic peptide corresponding to the C-terminal region of the deduced wheat CDC2 protein sequence. Following immunisation, rabbit antisera exhibited a strong immune response against the synthetic peptide, as demonstrated by ELISA analysis (Figure 1).

**Figure 2:**
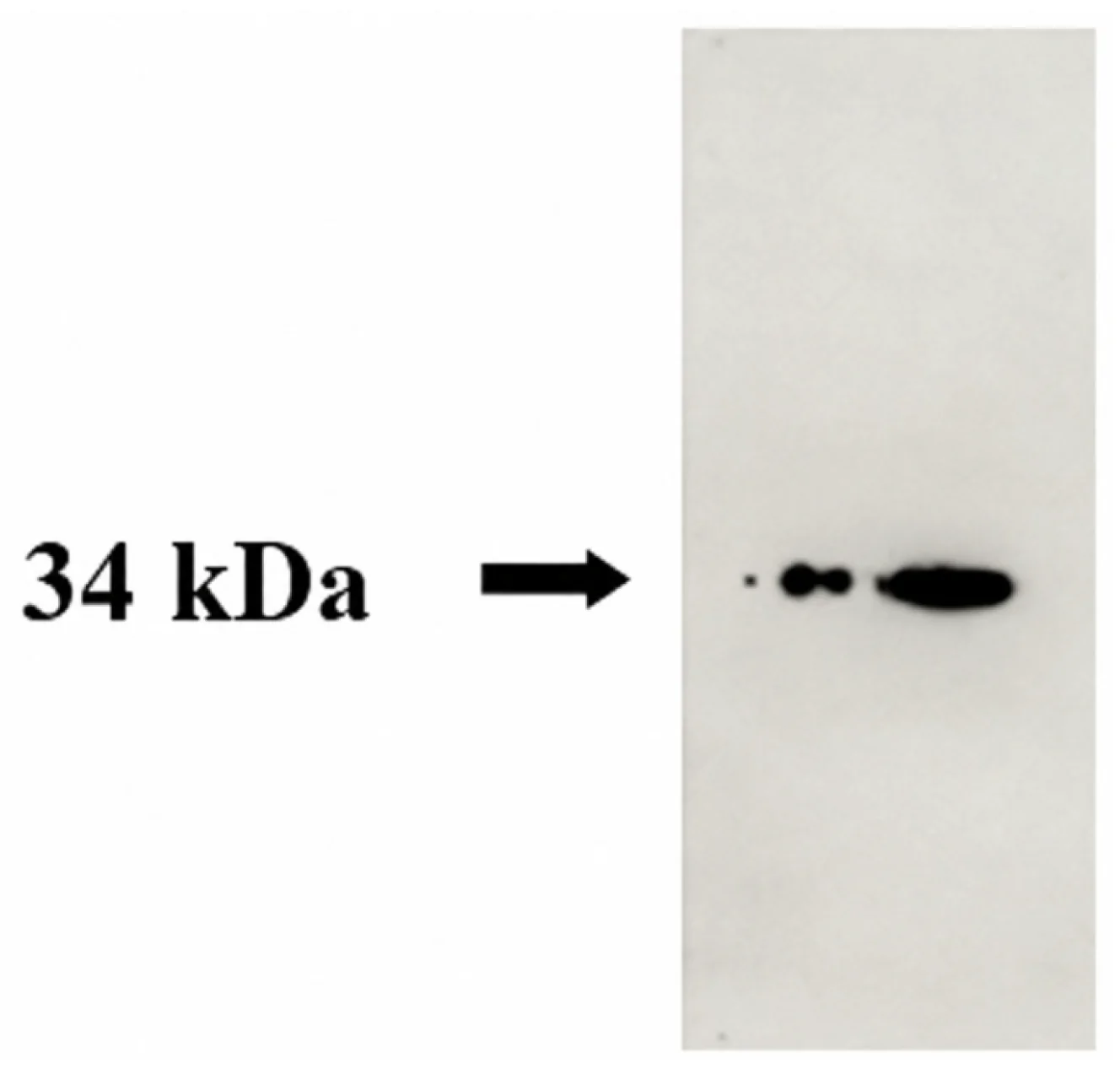
Immunoblot of total protein from fusion products of wheat gametes probed with CDC2 antibody to check the specificity of the Ab following optimisation of the Western Blotting protocol. With optimised parameters, the antibody reveals a single band depicting a protein of 34 kDa.

The specificity of the affinity-purified antibody was evaluated by immunoblotting. A single immunoreactive band with an apparent molecular mass of approximately 34 kDa was consistently detected in protein extracts prepared from fertilised wheat egg cells (Figure 2).

The purified Ab recognised a protein of 34 kD molecular weight on the immunoblot membrane, thus confirming that the antibody recognised the expected antigen and demonstrating that the affinity-purified antibody specifically recognised the wheat p34^cdc2^.

**Figure 3:**
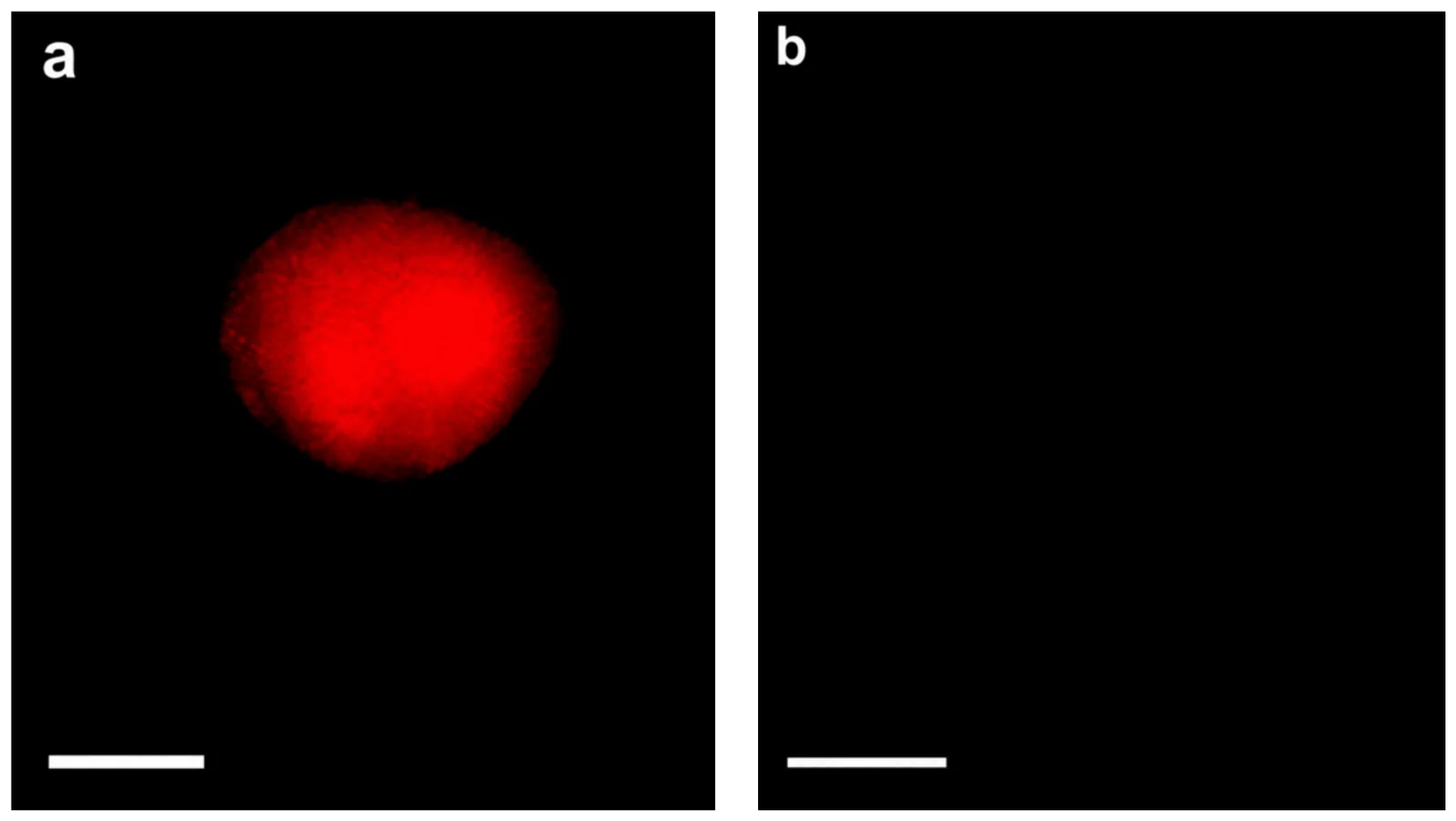
Fluorescence image of an in vitro-fertilised wheat egg cell (**a**) incubated with CDC2 antibody following fixation at 4 h after electrofusion with the sperm cell, showing a homogeneous staining pattern in the nucleus of the interphase cell. Primary antibody: wheat cdc2-specific antibody; secondary antibody: goat anti-rabbit antibody conjugated to Texas Red; (**b**) Negative control. Scale bar = 20 µm.

In interphase egg cells, p34^cdc2^-specific immunofluorescence appeared predominantly associated with the nucleus (Figure 3a), whereas no specific signal was detected in control experiments in which the primary antibody was omitted (Figure 3b), hence corroborating the specificity of the staining procedure.

### 2.2. Representative Embryo Development Following Re-Implantation into Maternal Tissues

**Figure 4:**
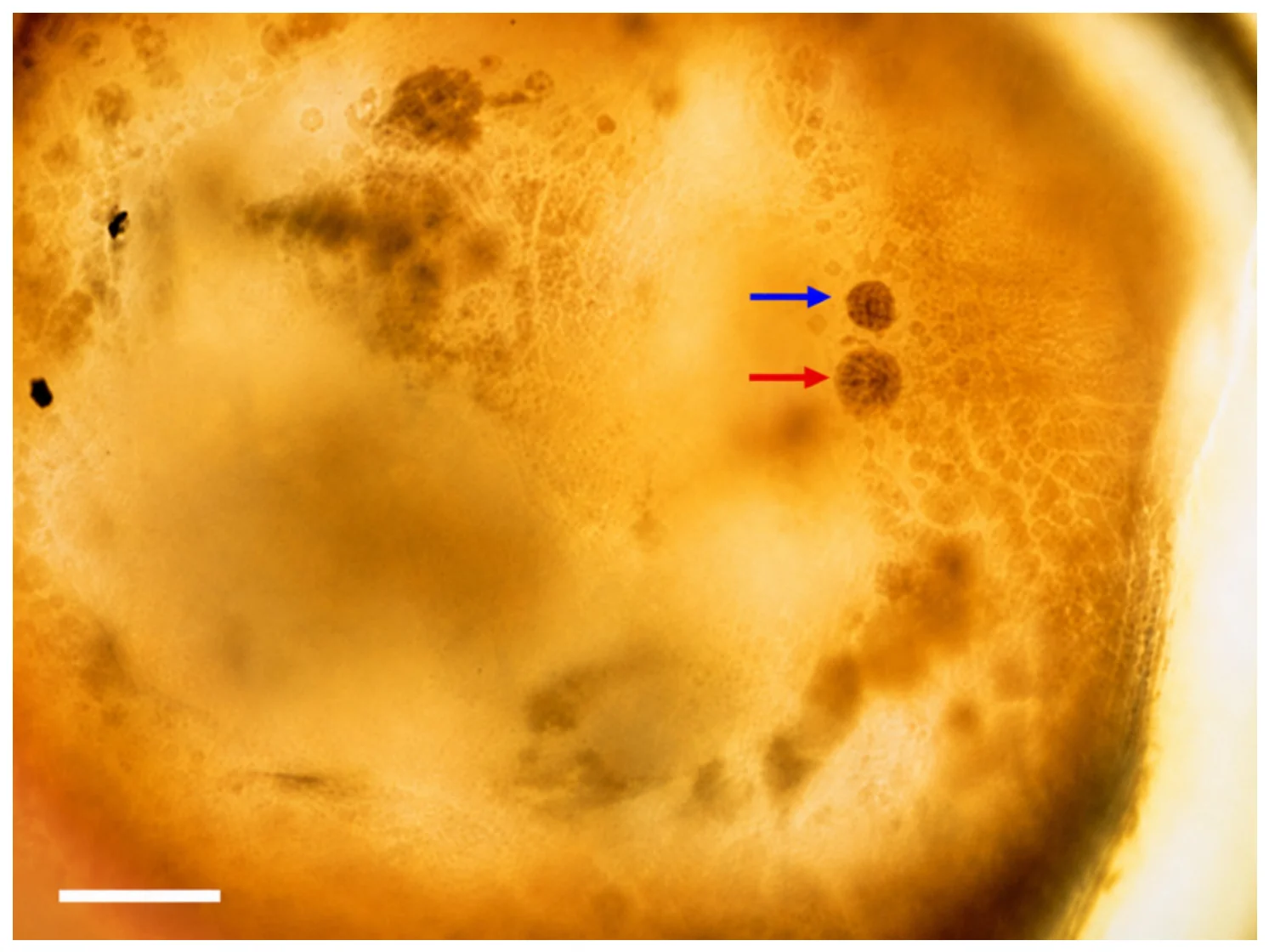
Micrograph of an egg cell re-implanted into an ovule following IVF. The blue arrow indicates the in vivo-fertilised egg cell in an ovule half, whereas the red arrow indicates an in vitro-fertilised egg cell introduced into the bisected ovule using a microcapillary mounted on a micromanipulator. Scale bar = 100 µm.

To examine development in the presence of surrounding maternal ovular tissues, in vitro-fertilised egg cells were re-introduced into dissected ovule tissues and cultured under semi-in vivo conditions. This experimental system permitted observation of re-implanted zygotes within an ovular environment (Figure 4); the present series does not quantify the effects of maternal cues.

**Figure 5:**
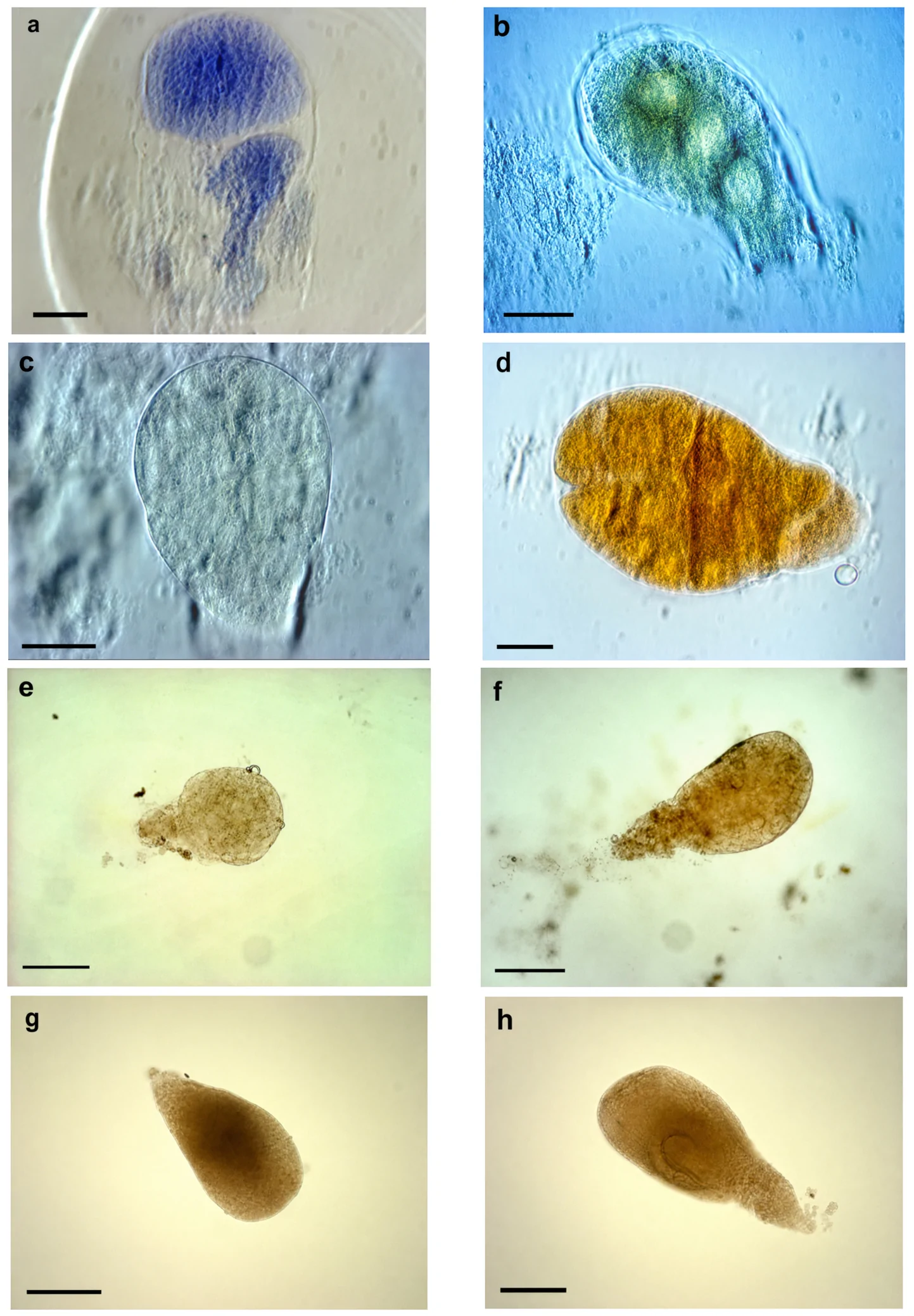
Representative developmental stages of wheat embryos derived from isolated egg cells fertilised in vitro and subsequently re-introduced into maternal ovular tissues. (**a**) Excised two-celled proembryo consisting of apical and basal cells, recovered 28 h after fertilisation (HAF). (**b**) Early globular embryo at 36 HAF. (**c**) Intermediate globular embryo excised at 48 HAF, showing extensive cell proliferation. (**d**) Late globular embryo isolated 3 days after fertilisation (DAF). (**e**) Transition-stage embryo isolated 6 DAF. (**f**) Early pear-shaped embryo isolated 9 DAF. (**g**) Advanced pear-shaped embryo excised 15 DAF. (**h**) Coleoptilar-stage embryo isolated 18 DAF following in vitro fertilisation and re-implantation into maternal ovular tissues. The panels depict different specimens collected at the indicated times; stage-specific frequencies were not determined. Scale bars: (**a**,**b**) = 10 µm; (**c**,**d**) = 20 µm; (**e**,**f**) = 150 µm; (**g**,**h**) = 200 µm. Abbreviations: HAF, hours after fertilisation; DAF, days after fertilisation.

Following re-implantation, individual fertilised egg cells were observed at developmental stages resembling those described during normal in vivo embryogenesis [62,63]. The examples illustrated in Figure 5 comprise a two-celled proembryo at 28 h after fertilisation (Figure 5a), an early globular embryo at 36 h (Figure 5b), intermediate and late globular forms at 48 h and 3 days, respectively (Figure 5c,d), and later transition, pear-shaped and coleoptilar-stage embryos (Figure 5e–h). These images document stages observed at the indicated sampling times; they do not determine the frequency with which re-implanted zygotes reached each stage or demonstrate that the same embryo progressed through the entire series.

The representative embryos illustrated in Figure 5 show that some in vitro-fertilised wheat egg cells were capable of attaining organised embryonic stages after re-implantation into maternal ovular tissues. These observations are consistent with the possibility that the ovular environment supports embryogenic development and polarity maintenance. However, because reliable stage-specific frequencies and a quantitative comparison with isolated culture are unavailable for this series, the images cannot establish the proportion of re-implanted zygotes retaining embryogenic competence or demonstrate that maternal signals are required for normal pattern formation. The semi-in vivo system therefore provides a descriptive experimental framework for examining early wheat embryogenesis and for testing the contribution of maternal cues in future quantitative studies.

### 2.3. Stage-Associated Differences in p34cdc2 Localisation Before the First Zygotic Division

**Figure 6:**
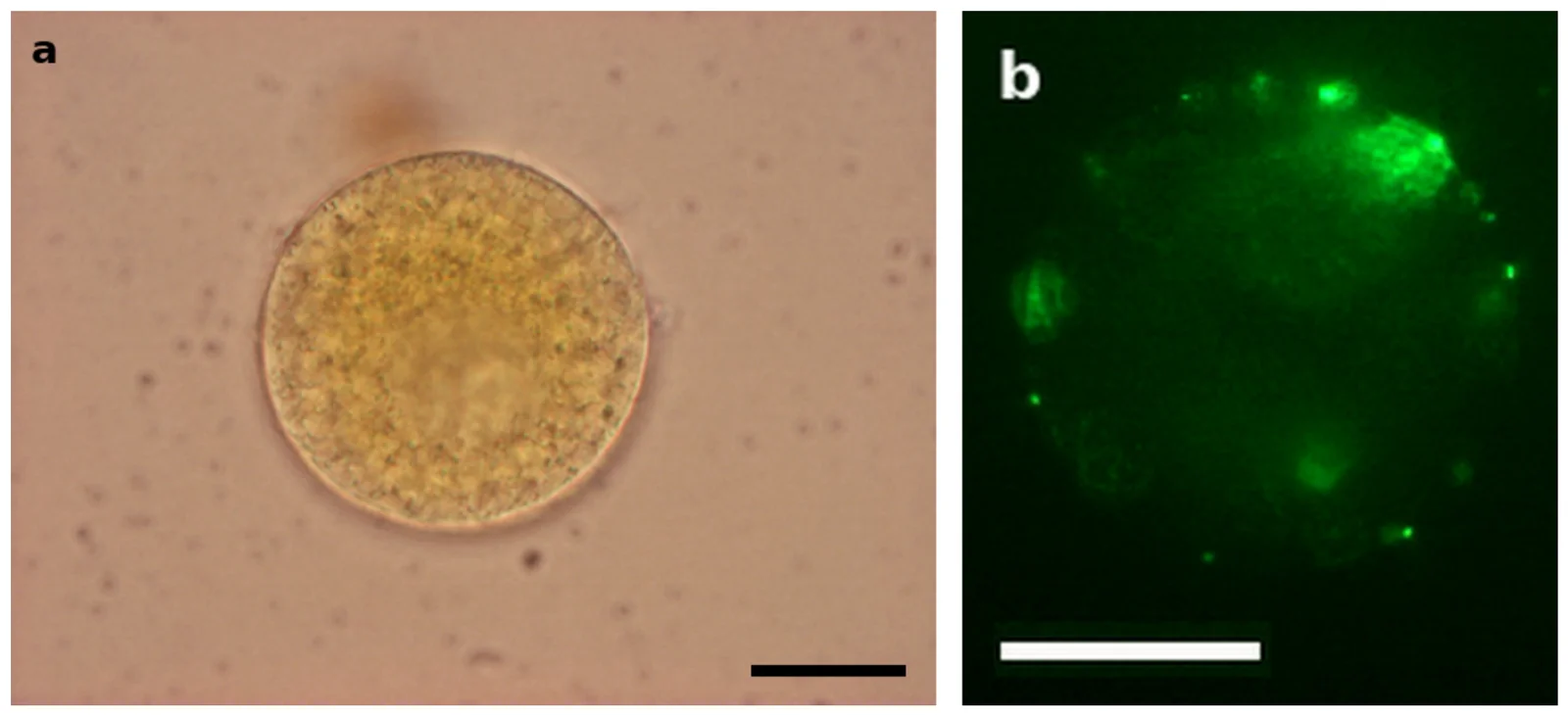
Visualisation of microtubules in a fertilised wheat egg cell by immunostaining with an α-tubulin-specific monoclonal antibody. (**a**) Bright-field image and (**b**) corresponding fluorescence image of the same egg cell. Note the intense patch-like fluorescence in the subcortical region. The panels are presented at different image scales. Scale bars = 20 µm.

To investigate whether p34cdc2 localisation changes during the developmental interval preceding the first embryogenic division, fertilised egg cells were analysed at successive stages following sperm–egg fusion. Because immunolocalisation required fixation, the illustrated time points represent different zygotes rather than time-lapse observation of the same cell. The frequency of each localisation pattern was not determined systematically. In unfertilised egg cells, tubulin was localised predominantly within the cortical cytoplasm and in cytoplasmic strands extending towards the nucleus (Figure 6).

**Figure 7:**
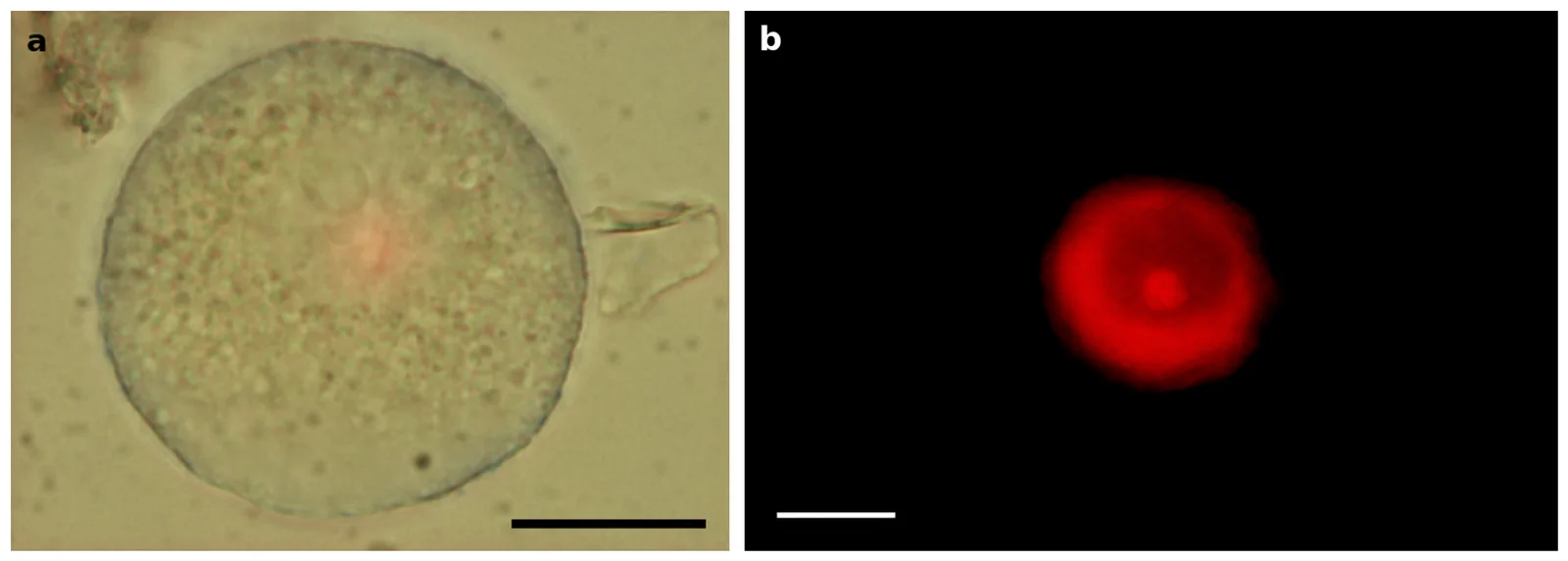
Spatio-temporal localisation of p34cdc2 in a fertilised wheat egg cell 4 h after sperm–egg fusion. (**a**) Bright-field image and (**b**) corresponding immunofluorescence image of the same egg cell. The images were acquired at higher magnification using a 100× oil-immersion objective to provide a clearer view of the nuclear signal. p34cdc2 fluorescence is concentrated in the nucleolar region and shows an asymmetric crescent-like distribution within the nucleus. Primary antibody: affinity-purified anti-p34cdc2; secondary antibody: Texas Red-conjugated goat anti-rabbit IgG. Scale bars = 20 µm.

Four hours after fertilisation, the illustrated zygote exhibited a markedly asymmetric p34cdc2 localisation pattern within the nucleus (Figure 7).

**Figure 8:**
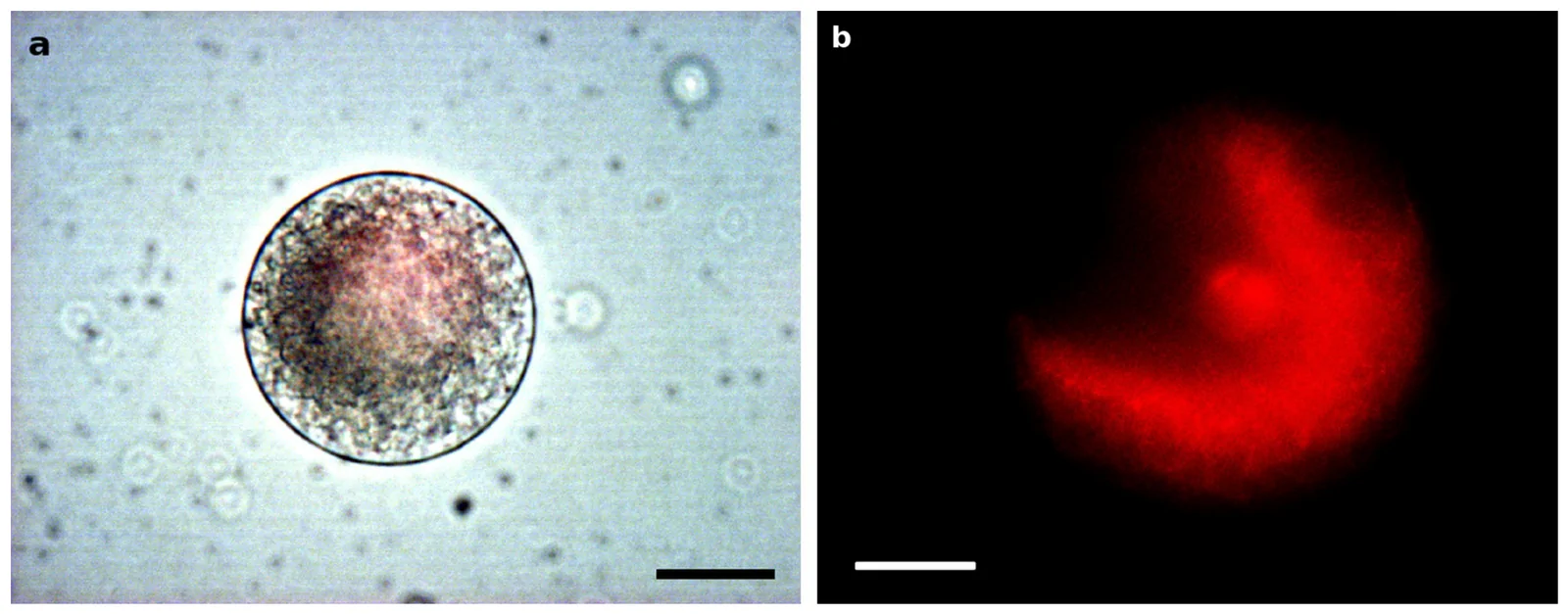
Redistribution of p34cdc2 in a fertilised wheat egg cell 6 h after sperm–egg fusion. (**a**) Bright-field image and (**b**) corresponding immunofluorescence image of the same egg cell. Compared with the earlier nuclear localisation, the p34cdc2 signal is redistributed from the nucleus towards the surrounding cytoplasm. The panels are presented at different image scales. Scale bars = 20 µm.

Rather than being distributed uniformly, the protein accumulated preferentially within one region of the nucleus, producing a pronounced crescent-like fluorescence pattern. The strongest signal was observed to be associated with the nucleolar region. In the zygote examined 6 h after fertilisation, the fluorescence signal appeared less restricted to the nucleus and increasingly appeared within the surrounding cytoplasm (Figure 8).

**Figure 9:**
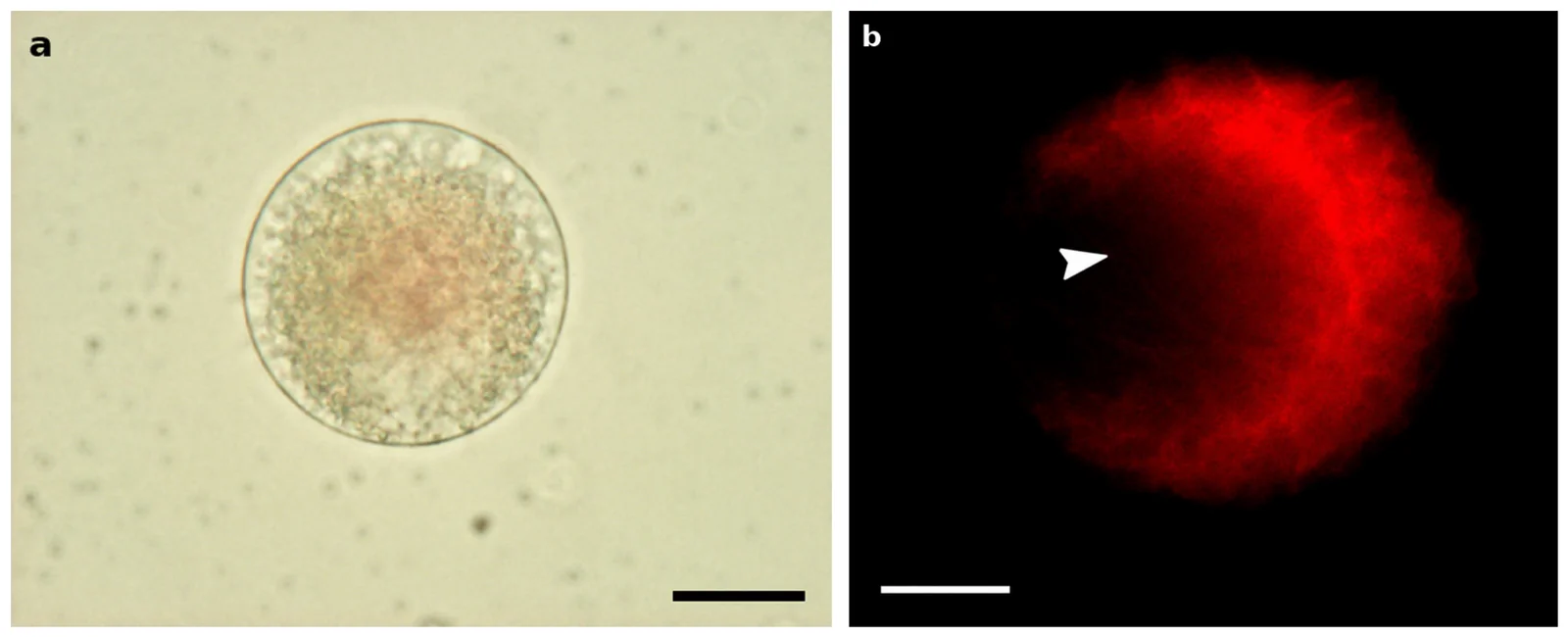
Predominantly cytoplasmic localisation of p34cdc2 in a fertilised wheat egg cell 8 h after sperm–egg fusion. (**a**) Bright-field image and (**b**) corresponding immunofluorescence image of the same egg cell. At this stage, the p34cdc2 signal is predominantly cytoplasmic, with little detectable fluorescence in the nuclear region. The panels are presented at different image scales. Scale bars = 20 µm. The arrowhead points to the nuclear region.

In the specimen examined 8 h after fertilisation, p34cdc2 fluorescence was predominantly cytoplasmic, with little detectable nuclear signal (Figure 9). However, this observation should not be interpreted as direct evidence of progressive translocation within a single zygote.

The fixed-cell images illustrate distinct p34cdc2 localisation patterns at selected stages preceding the first zygotic division, including nuclear, asymmetrically nuclear and predominantly cytoplasmic distributions. These patterns were observed at times overlapping the interval of zygotic polarisation. Because the time points represent different fixed cells and pattern frequencies were not determined, the series does not establish the prevalence or obligatory temporal order of these patterns, nor does it demonstrate protein translocation within an individual zygote.

### 2.4. Association of p34cdc2 with the Preprophase Band During Division-Plane Specification

**Figure 10:**
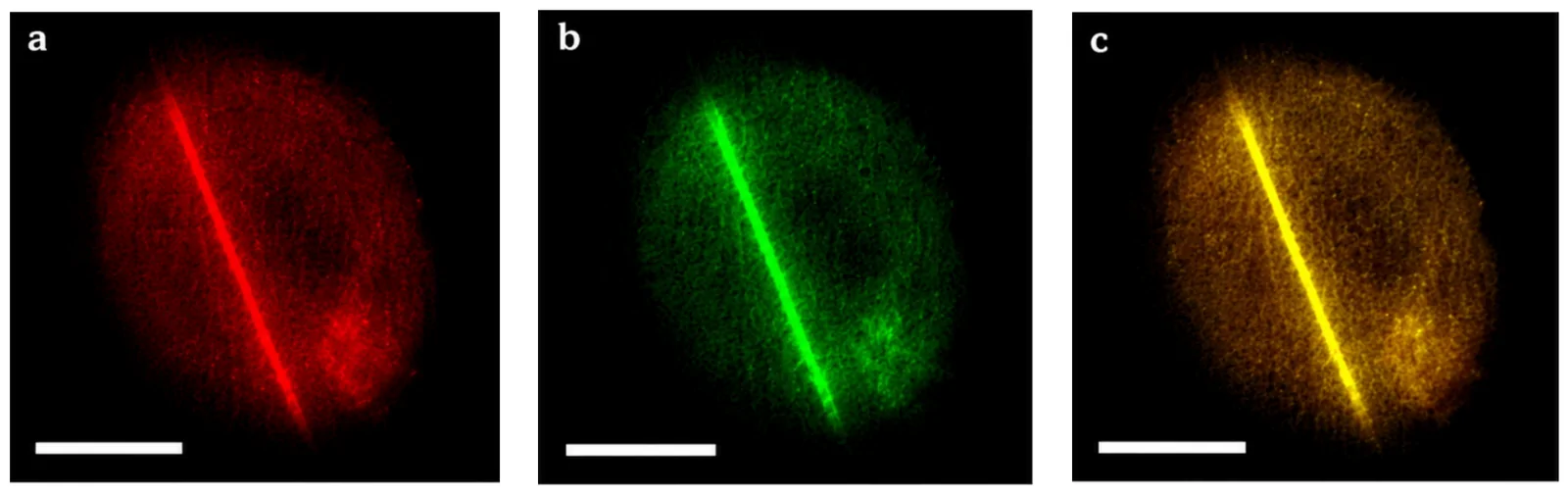
Co-localisation of p34cdc2 and microtubules in the preprophase band in an in vitro-fertilised egg cell. (**a**) Immunolocalisation of p34cdc2 (red). (**b**) Microtubules visualised with an anti-α-tubulin antibody (green). (**c**) Merged image showing co-localisation (yellow) of both signals in the preprophase band. The distinct cortical array corresponds to the preprophase band that defines the orientation of the future division plane. Scale bars = 20 µm.

A remarkable localisation pattern could be observed in the zygote in an example showing the p34cdc2 signal associated with the preprophase band (PPB), a plant-specific microtubular structure that predicts the future cell division plane. Double-labelling experiments revealed substantial overlap between p34cdc2 and tubulin fluorescence within the PPB region of prophase zygotes (Figure 10).

**Figure 11:**
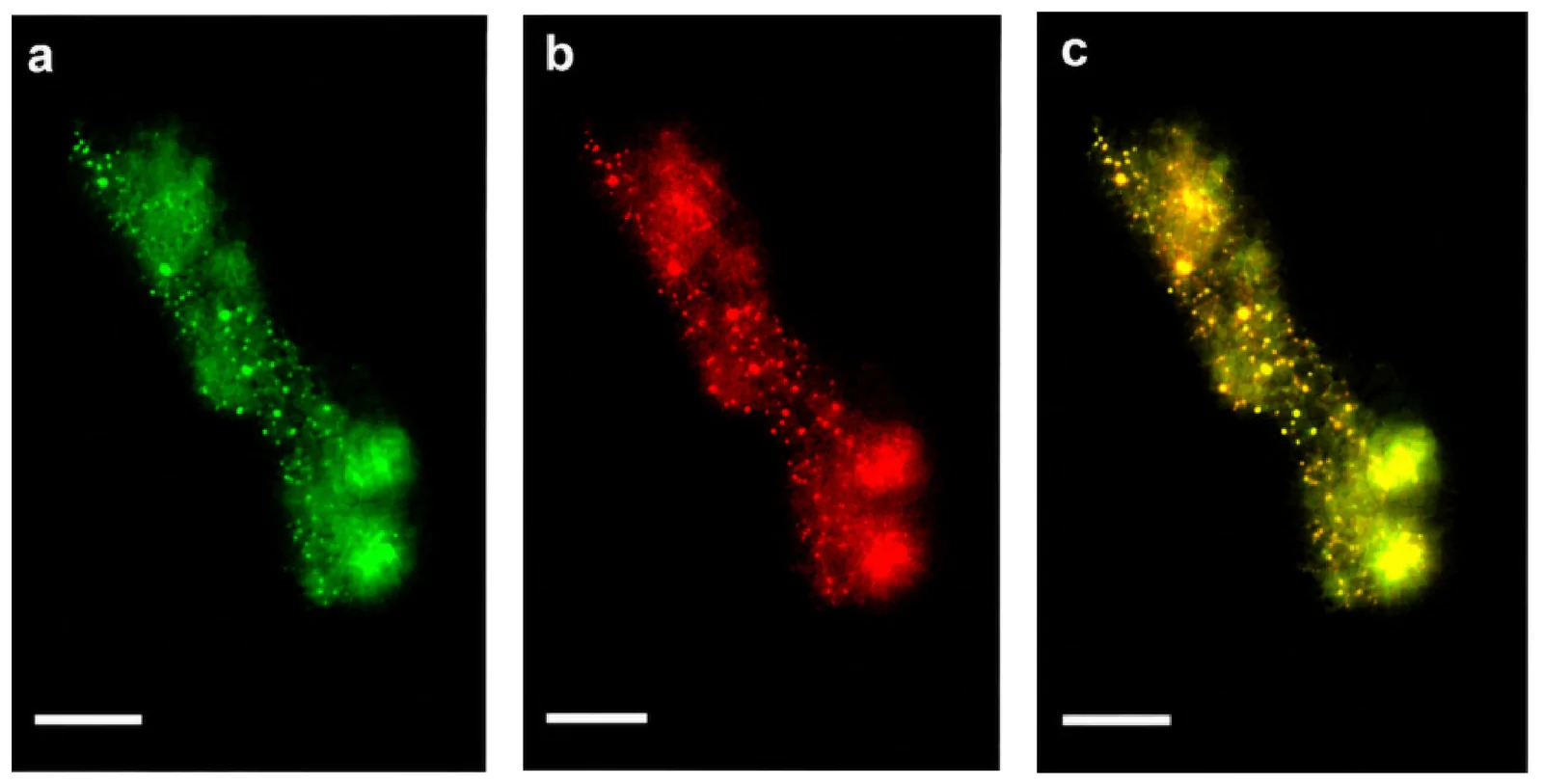
Co-localisation of p34^cdc2^ with the preprophase band (PPB) in an early prophase wheat zygote. (**a**) Microtubules visualised with an anti-α-tubulin antibody (green). (**b**) Immunolocalisation of p34cdc2 (red). (**c**) Merged image demonstrating the co-localisation (yellow) of both signals, showing the preferential accumulation of p34^cdc2^ at the cortical preprophase band. Scale bars = 15 µm.

Merged fluorescence images showed close spatial coincidence between the kinase and the cortical microtubule array defining the future division site. Confocal microscopy provided a further image consistent with this observation and showed that the p34cdc2 signal prevailed at the site of the PPB (Figure 11).

**Figure 12:**
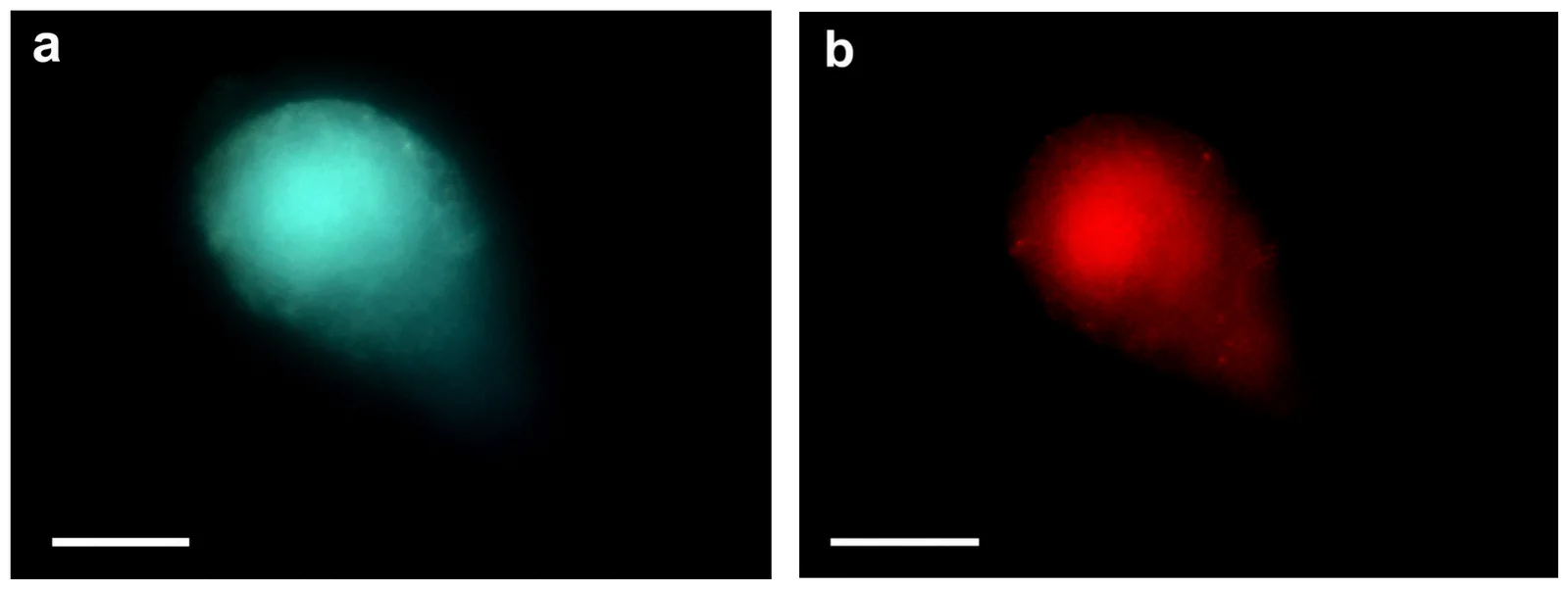
Nuclear localisation of p34cdc2 in a polarised wheat zygote. (**a**) Nuclear visualisation via DAPI staining. (**b**) Immunodetection of p34cdc2 using an affinity-purified anti-CDC2 antibody. The strong fluorescent signal, localised within the nucleus of this polarised cell, confirms the nuclear retention of p34cdc2 at this developmental stage. Scale bars = 20 µm.

**Figure 13:**
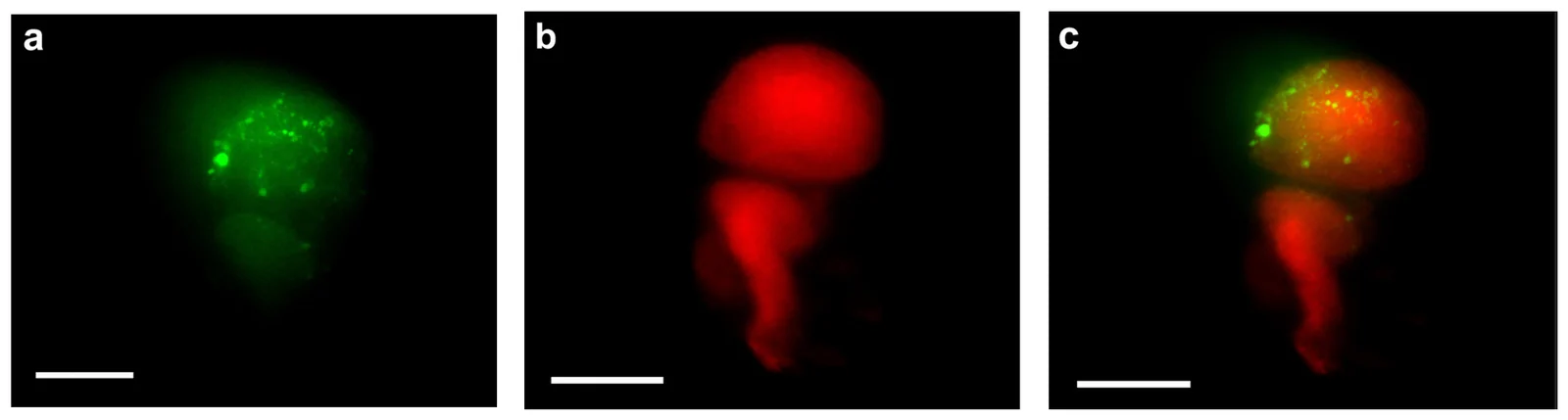
Double-immunolabelling of microtubules and p34cdc2 in proembryos excised 36 h after re-implantation. (**a**) Microtubule localisation visualised with an anti-α-tubulin antibody (green); (**b**) p34cdc2 distribution (red); (**c**) merged image. Intense p34cdc2 staining is evident in the nucleolus of the apical cell and throughout the cytoplasm of both daughter cells. Microtubule staining appears spatially restricted to a distinct pole of the apical cell, as highlighted in the merged image (**c**). Scale bars = 50 µm.

**Figure 14:**
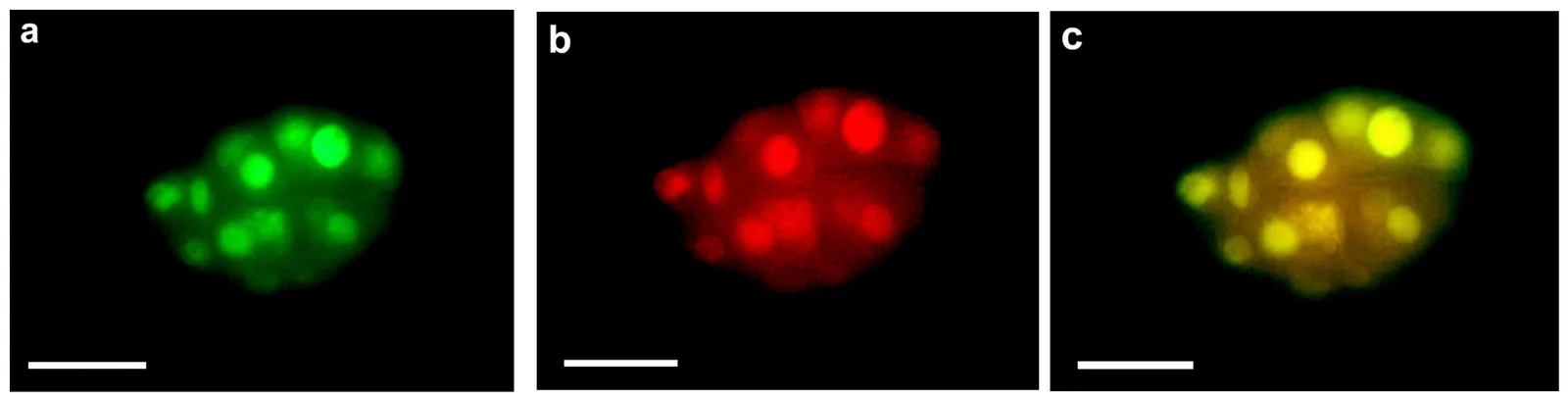
Nuclear localisation of p34cdc2 in a multicellular proembryo following a 6-day ‘nurse culture’ period within maternal tissues. (**a**) Embryo stained with SYTO-Green to visualise nuclei (green); (**b**) immunodetection of p34cdc2 (red); (**c**) merged image showing intense co-localisation (yellow) of the p34cdc2 signal within the nuclei. This indicates robust nuclear accumulation of p34cdc2 at this stage of embryogenesis. Scale bars = 100 µm.

The highly ordered distribution of p34cdc2 contrasted markedly with the more diffuse patterns observed during earlier developmental stages. As the PPB represents one of the earliest cytological manifestations of spatial information within a dividing plant cell, localisation of p34cdc2 to this structure suggests that CDC2 activity is associated with mechanisms controlling division-plane specification before the onset of mitosis. As development progressed towards cytokinesis, p34cdc2 remained preferentially associated with the nucleus-containing cytoplasmic domain of the zygote (Figure 12). Following asymmetric division, strong fluorescence was detected in both daughter cells, although signal intensity was higher within the apical cell in the illustrated specimen (Figure 13). In more advanced embryos, p34cdc2 remained closely associated with nuclei of actively proliferating embryonic cells (Figure 14). Taken together, these representative images are consistent with stage-associated differences in p34cdc2 localisation, but the absence of pattern-frequency data precludes conclusions about how consistently these distributions occur during embryogenesis.

### 2.5. Distribution of p34cdc2 During Proembryo Formation and Early Embryogenesis

To determine whether the different localisation patterns illustrated during zygote development persisted after the first embryogenic division, p34cdc2 distribution was examined in young proembryos and developing embryos. Prior to cytokinesis, p34cdc2 was seen to be accumulated predominantly within that cytoplasmic domain which contained the nucleus (Figure 12).

The opposite pole of the zygote displayed substantially weaker fluorescence, resulting in a pronounced intracellular asymmetry. The illustrated polarised zygote displayed this localisation pattern. Following cytokinesis, p34cdc2 remained detectable in both daughter cells of the two-celled proembryo (Figure 13).

Fluorescence was distributed throughout the cells but was particularly intense in the illustrated cells in nuclear regions. A particularly intense signal was visible within the nucleolus of the apical daughter cell. Double-labelling experiments showed overlap between p34cdc2 and microtubular structures during this developmental stage. As embryogenesis progressed, p34cdc2 remained closely associated with nuclei of actively proliferating embryonic cells (Figure 14).

Strong fluorescence was detected in regions exhibiting intense mitotic activity, whereas weaker signals were generally associated with more differentiated tissues. These representative observations are consistent with an association between p34cdc2 localisation and proliferative embryonic regions after the first asymmetric division, but do not quantify the frequency of these patterns. The preferential localisation of the kinase within the apical lineage further suggests a relationship between CDC2-associated processes and developmental pattern formation during early embryo development.

### 2.6. p34cdc2 Is Detectable Throughout Early Embryogenesis

**Figure 15:**
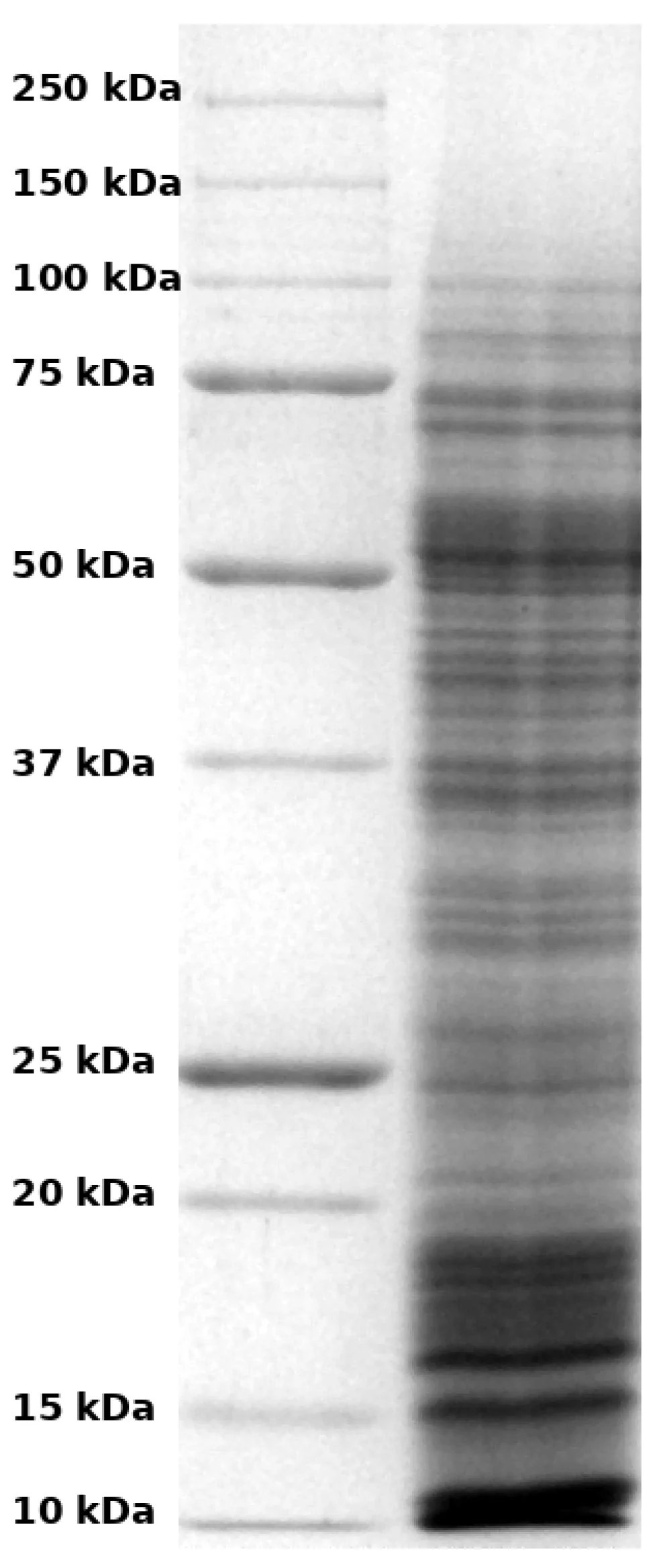
SDS-PAGE analysis of total protein extracted from in vitro-fertilised wheat egg cells. Total protein samples were resolved on a 12.5% SDS-polyacrylamide gel. The left lane contains molecular-mass standards (in kDa).

To establish whether developmental regulation involved not only dynamic changes in p34cdc2 localisation, but also changes in overall protein abundance, protein extracts prepared from fertilised egg cells collected at successive developmental stages were analysed by immunoblotting (Figure 15).

**Figure 16:**
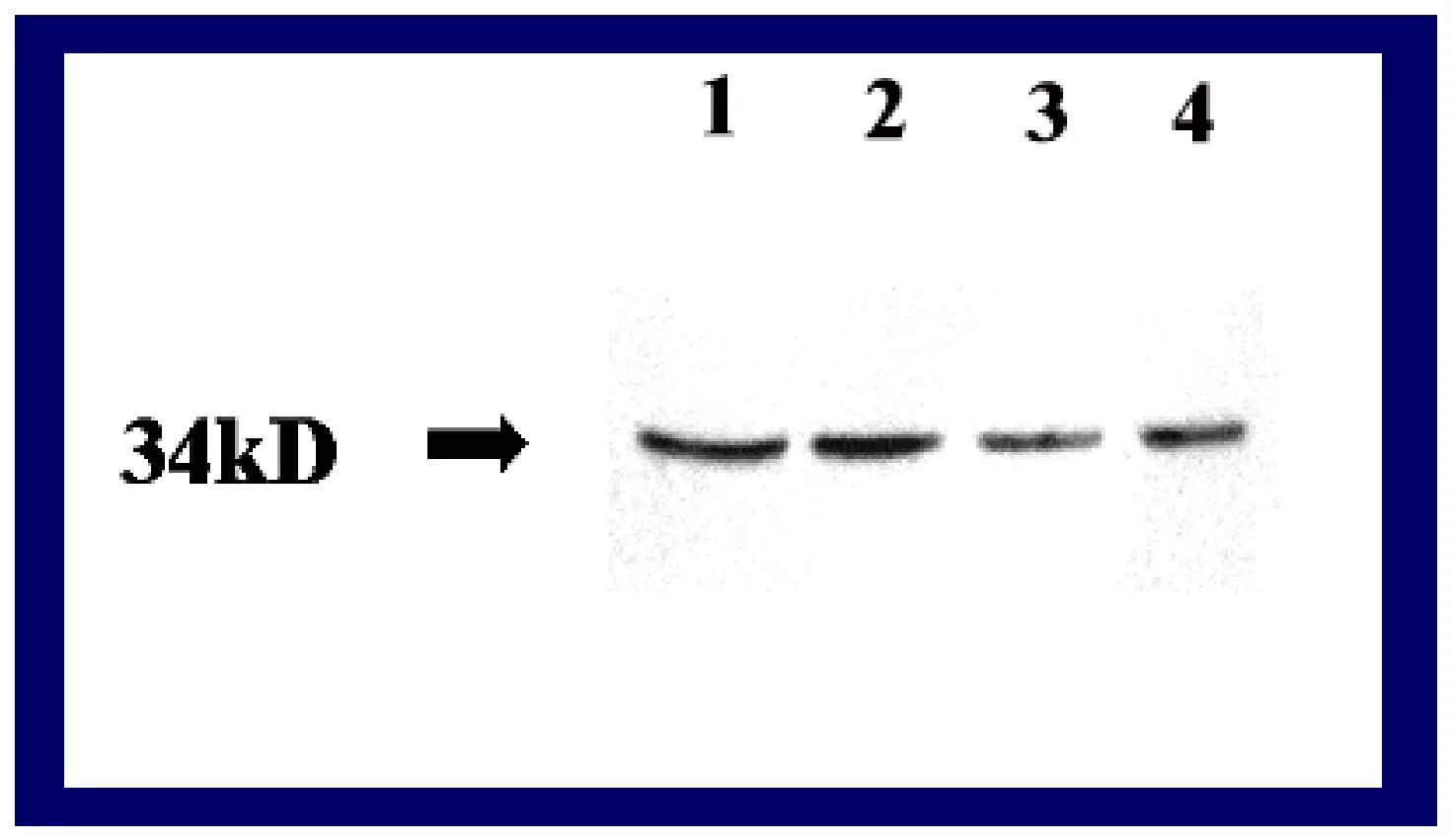
Immunoblot analysis of p34cdc2 in in vitro-fertilised wheat egg cells collected at defined time intervals following sperm–egg fusion. Total protein was extracted from fertilised egg cells harvested 8 h (lane 1), 12 h (lane 2), 16 h (lane 3), and 20 h (lane 4) after in vitro fertilisation. Immunodetection was performed using the affinity-purified anti-CDC2 antibody. A single immunoreactive band corresponding to approximately 34 kDa was detected at all examined time points, suggesting the continued presence of p34cdc2 during early zygotic development.

Protein extracts obtained from zygotes harvested 8, 12, 16, and 20 h after fertilisation revealed a detectable band corresponding to p34cdc2 (Figure 16).

No major qualitative differences in the immunoblot signal were apparent among the stages examined. Taken together, these results suggest that p34cdc2 was detectable throughout the developmental interval examined. Consequently, the illustrated differences in immunolocalisation cannot readily be explained by an obvious loss of detectable protein. Instead, they are compatible with differences in subcellular distribution; the present experiments do not directly measure kinase activation or post-translational regulation.

### 2.7. Disruption of the Microtubular Cytoskeleton Prevents Localisation of p34cdc2 to the Future Division Plane

**Figure 17:**
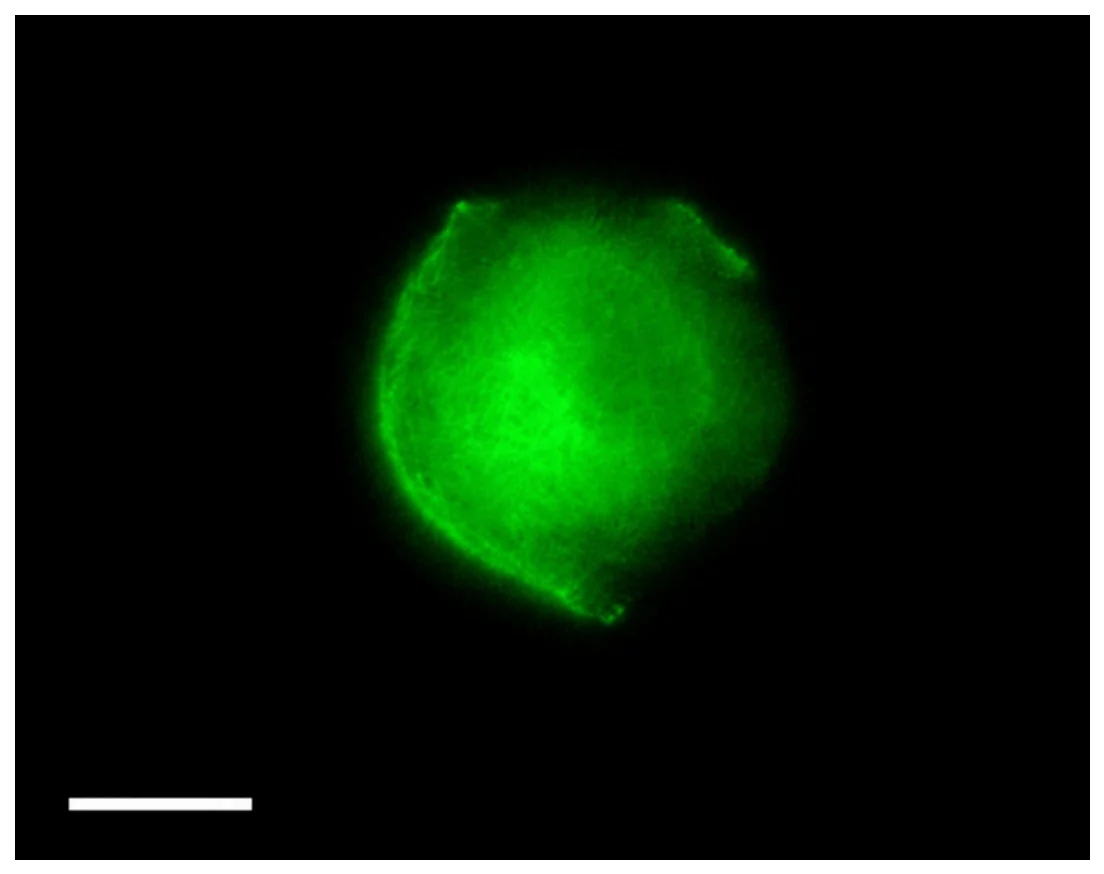
Oryzalin-treated, fertilised wheat egg cells in early prophase stained with an anti-α-tubulin antibody. The brightly fluorescing cytoplasm reflects staining of depolymerised microtubules. Scale bar = 20 µm.

The close association between p34cdc2 and the preprophase band suggested that localisation of the kinase might depend upon the integrity of the microtubular cytoskeleton. To test this possibility, fertilised egg cells were treated with the microtubule-depolymerising agent, oryzalin. In untreated control cells, p34cdc2 exhibited the characteristic PPB-associated localisation pattern observed during prophase. In contrast, exposure to oryzalin resulted in complete disruption of the cortical microtubule array and abolished the distinct localisation of p34cdc2 within the PPB region (Figure 17).

In the treated cells, p34cdc2 displayed a diffuse intracellular distribution, and its accumulation at the future division site could not be observed any longer. The loss of PPB-associated localisation upon microtubule depolymerisation points to the need for an intact microtubular framework for proper positioning of p34cdc2. These observations support a presumptive, functional relationship between CDC2-associated processes and the cytoskeletal machinery playing a substantial role in cell division-plane determination. Since the determination of the future division plane is pivotal during the first embryogenic cell cycle, the association between p34cdc2 and the PPB suggests that CDC2-associated processes may be involved in those intricate developmental processes that link spatial organisation of cell components with cell-cycle progression.

### 2.8. CDK Inhibition and Antibody-Mediated Perturbation of CDC2 Function Reveal a Critical Developmental Window

**Figure 18:**
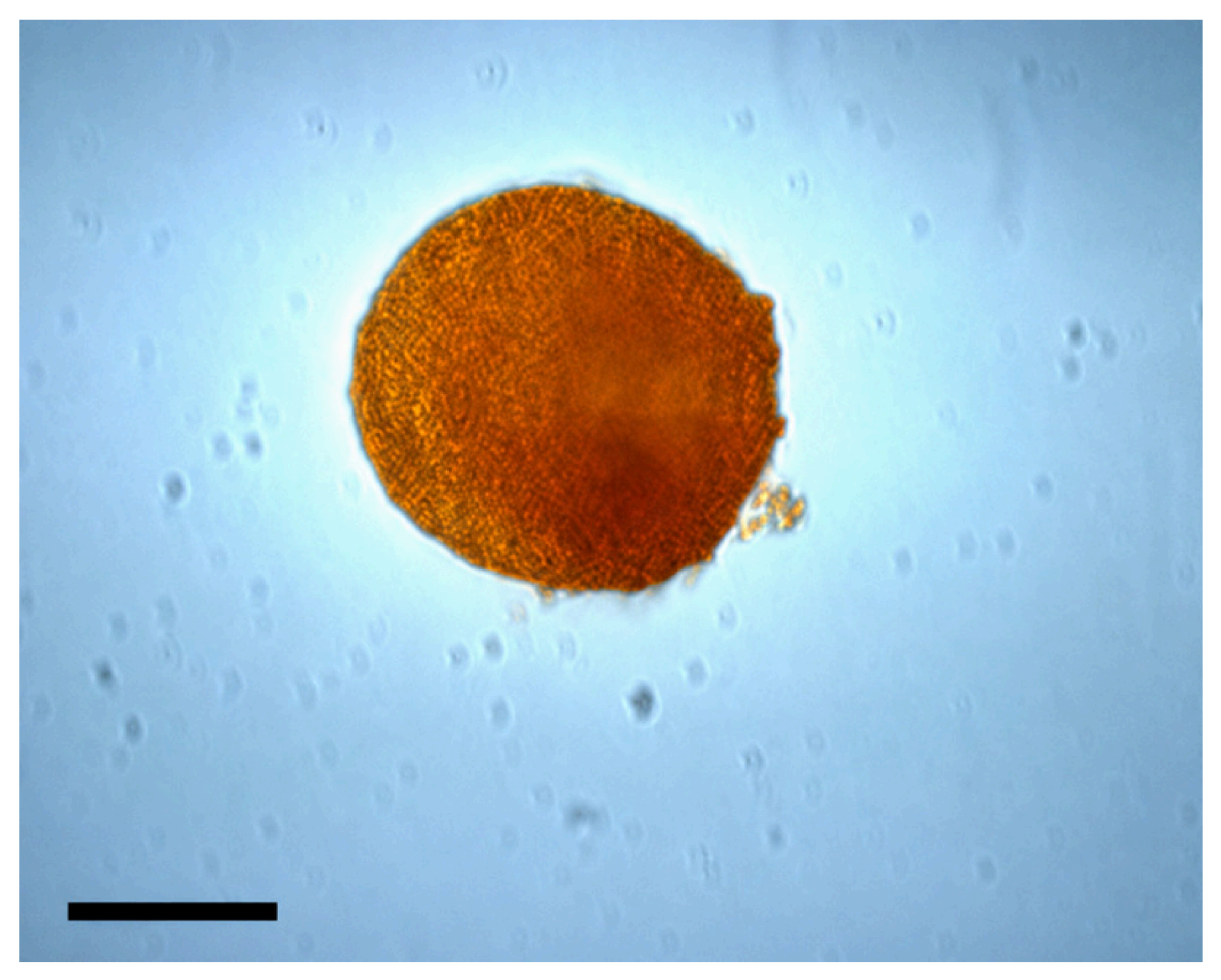
Fertilised wheat egg cell following treatment with 100 µM olomoucine. Olomoucine was applied 2 h after sperm–egg fusion (HAF). The treated cell remained undivided throughout the observation period and displayed no visible signs of normal embryogenic progression, consistent with interference with CDK-associated cell-cycle progression. Scale bar = 20 µm.

To investigate the putative roles of CDC2-associated processes during embryogenesis, fertilised egg cells were treated with drugs to induce pharmacological inhibition of cyclin-dependent kinases and subjected to antibody-mediated interference with CDC2 activity. Treatment with olomoucine led to a remarkable inhibition of embryonic development. Of 44 treated zygotes, 38 were seen to fail to divide, corresponding to an inhibition frequency of 86.4% (Figure 18).

**Figure 19:**
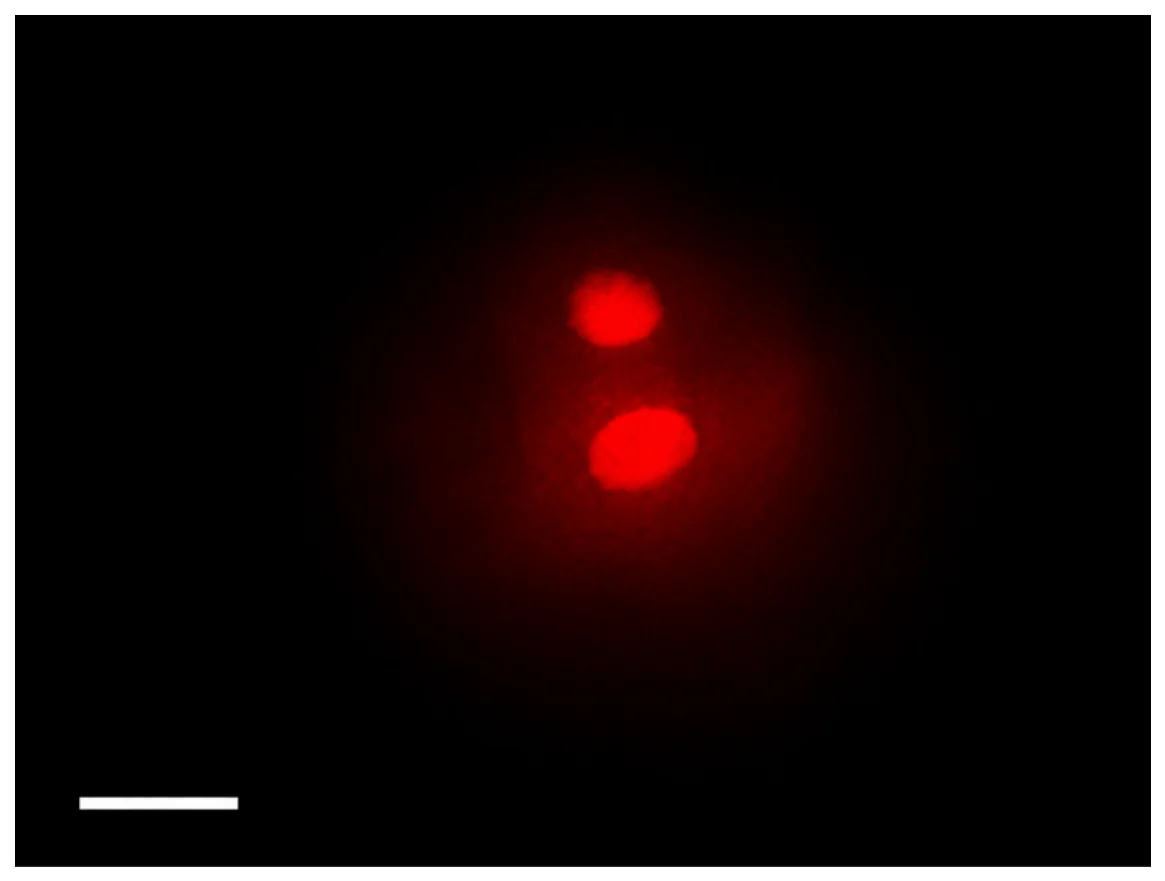
Division of an in vitro-fertilised wheat egg cell following treatment with isolomoucine, an inactive structural analogue of olomoucine. Isolomoucine was applied at a concentration of 400 µM. The two daughter nuclei were visualised by staining with the DNA-specific fluorescent dyeSYTOX Red (Molecular Probes, Eugene, OR, USA), indicating that cell division proceeded normally despite treatment. These observations support the specificity of the inhibitory effects observed with olomoucine. (Isolomoucine was applied at a fourfold-higher concentration because of its substantially lower inhibitory activity toward cyclin-dependent kinases.) Scale bar = 20 µm.

In contrast, 37 of 40 zygotes (92.5%) treated with the inactive structural analogue isolomoucine divided normally (Figure 19). The difference in division outcome between olomoucine- and isolomoucine-treated zygotes was significant (two-sided Fisher’s exact test, *p* < 0.001), supporting the specificity of the inhibitory effect observed with olomoucine.

**Figure 20:**
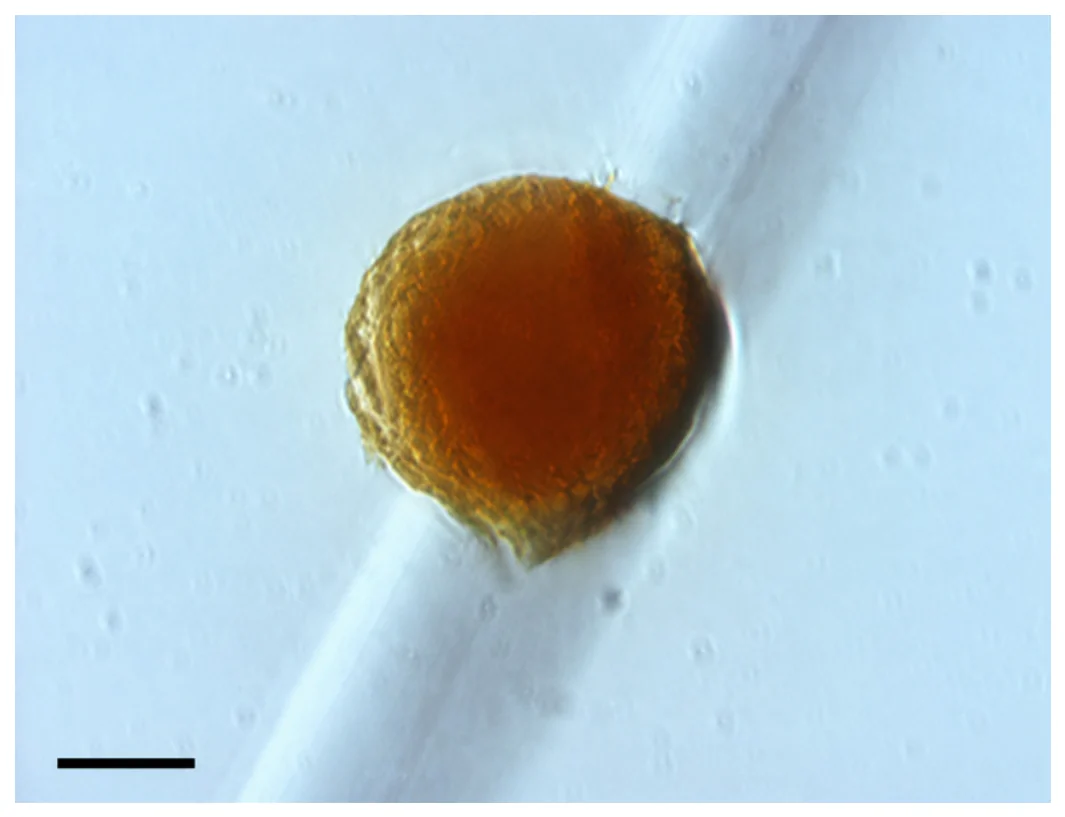
Fertilised wheat egg cell following treatment with roscovitine. Roscovitine (20 µM) was applied 2 h after sperm–egg fusion (HAF). Of 31 roscovitine-treated zygotes, 28 (90.3%) failed to divide. The cell shown remained undivided throughout the observation period and exhibited no visible signs of normal embryogenic progression, consistent with inhibition of CDC2-associated kinase activity and arrest of cell-cycle progression. Scale bar = 20 µm.

**Figure 21:**
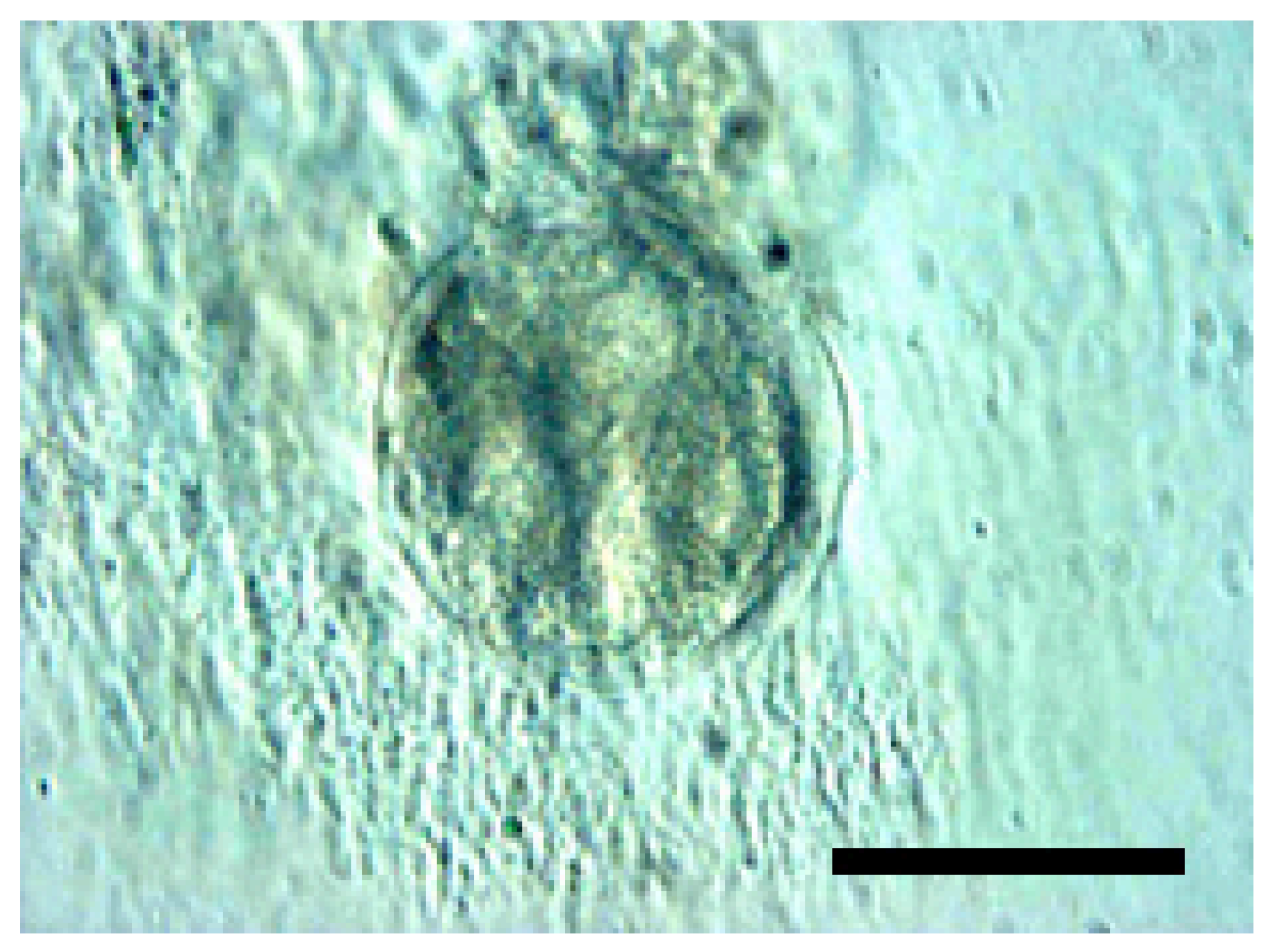
Fertilised wheat egg cell following treatment with amino-purvalanol. Amino-purvalanol was applied 2–4 h after sperm–egg fusion (HAF). Of 58 amino-purvalanol-treated zygotes, 42 (72.4%) failed to divide. Treated cells that remained undivided failed to undergo normal embryogenic progression, consistent with inhibition of CDC2-associated kinase activity and arrest of early zygotic development. Scale bar = 50 µm.

To determine whether the developmental arrest induced by olomoucine was consistent with CDK inhibition, additional pharmacological controls were performed using isolomoucine and roscovitine. The ability of two independent CDK inhibitors to induce similar phenotypes, together with the absence of an effect following treatment with the inactive analogue, supports involvement of CDK-sensitive processes, although off-target effects cannot be completely excluded. Comparable results were obtained with the CDK inhibitors roscovitine and amino-purvalanol (Figure 20 and Figure 21, respectively).

**Figure 22:**
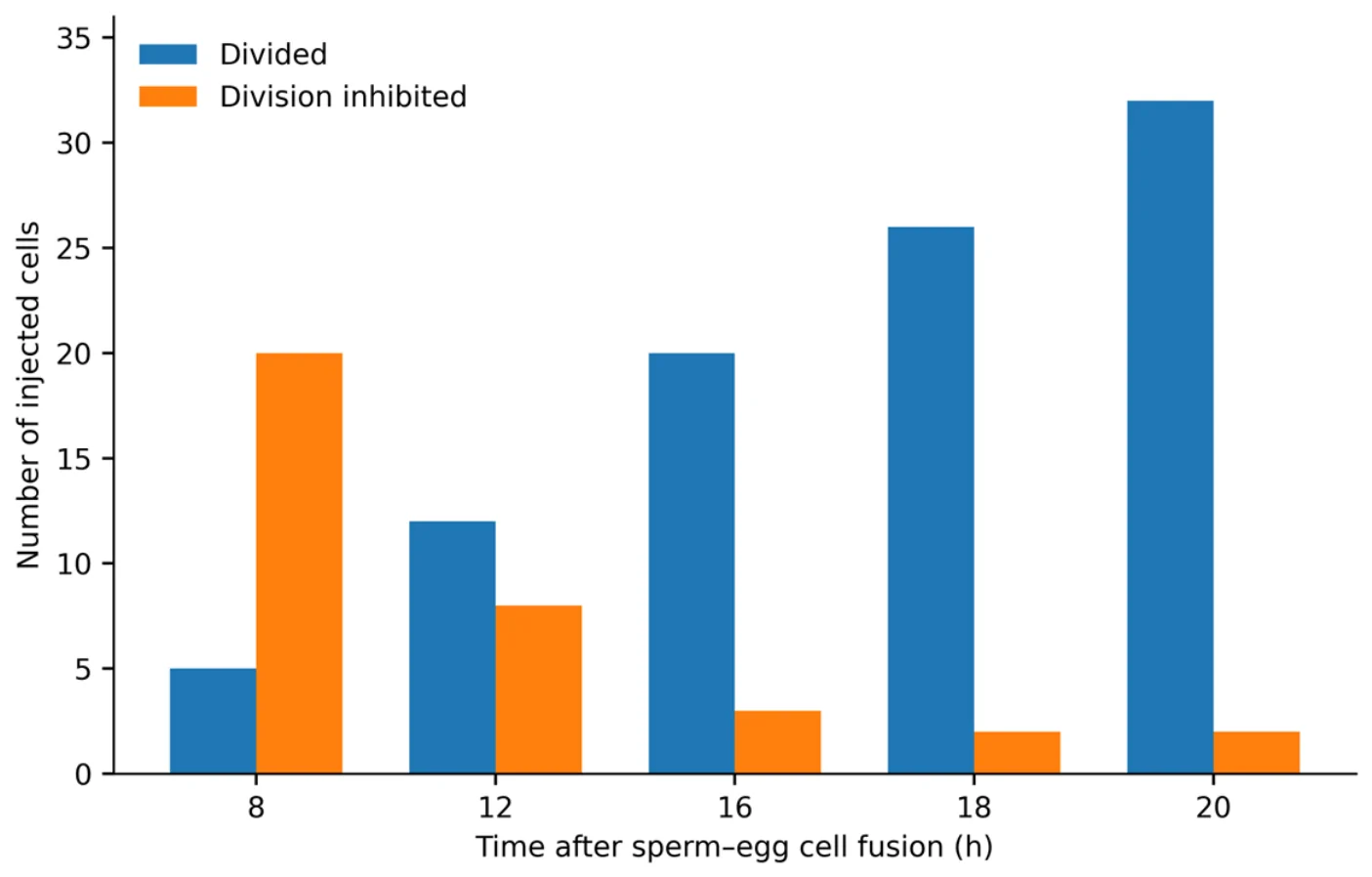
Effect of microinjection of the anti-CDC2 antibody on the division of in vitro-fertilised wheat zygotes. Microinjection performed 8 h after sperm–egg cell fusion strongly inhibited zygotic division, with 20 of 25 injected zygotes (80.0%) remaining undivided, whereas 23 of 25 buffer-injected control zygotes (92.0%) divided (two-sided Fisher’s exact test, *p* < 0.001). In contrast, microinjection at 20 h after fusion had little effect on developmental progression, allowing 32 of 34 injected zygotes (94.1%) to undergo cell division. These results indicate the existence of a critical early post-fertilisation period during which interference with CDC2-associated processes has a particularly strong effect on subsequent embryonic development.

**Figure 23:**
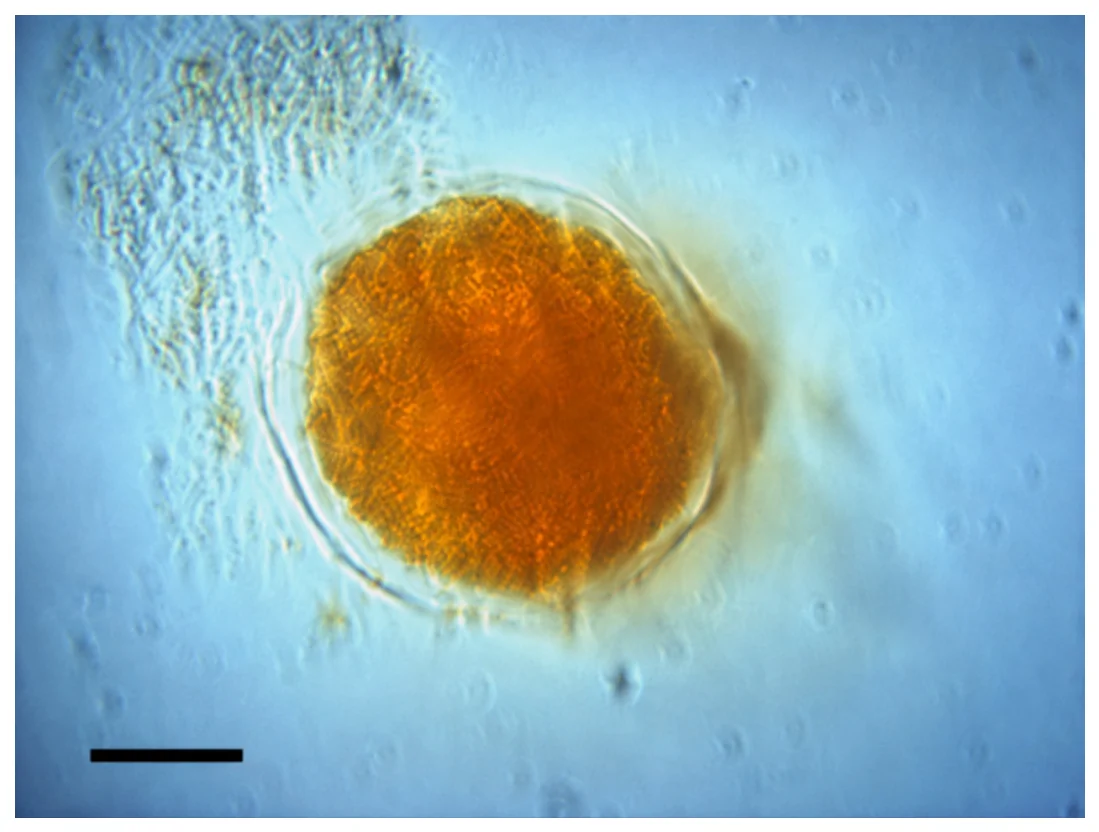
Bright-field micrograph of an in vitro-fertilised wheat egg cell microinjected with an anti-PSTAIRE antibody 2 h after sperm–egg fusion. Developmental arrest of an in vitro-fertilised wheat egg cell following microinjection of an anti-PSTAIRE antibody. The micrograph displays a cell microinjected 2 h after sperm–egg fusion. Overall, 22 of 23 anti-PSTAIRE-injected zygotes (95.7%) failed to divide. The cell shown remained morphologically intact but exhibited inhibition of progression through the first embryogenic cell cycle. Scale bar = 20 µm.

The consistent developmental responses observed with multiple structurally distinct inhibitors strongly support the assumption that CDK activity plays an important role during the first embryogenic cell cycle. Independent evidence for the involvement of CDC2-associated processes was obtained through antibody microinjection experiments. Fertilised egg cells injected with affinity-purified anti-CDC2 antibody displayed strong developmental inhibition, particularly when injections were performed shortly after fertilisation (Figure 22 and Figure 23).

**Figure 24:**
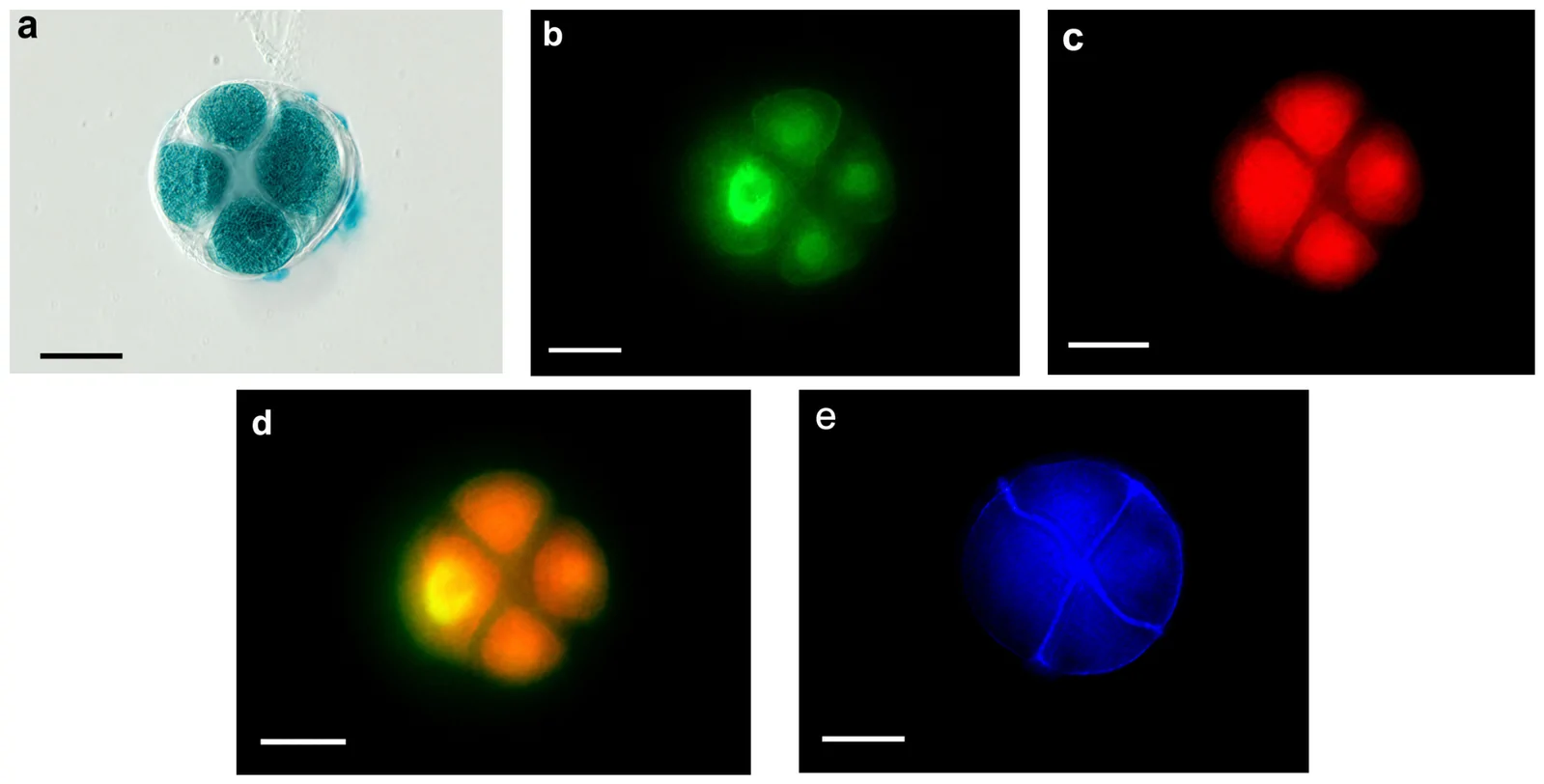
Aberrant division pattern in a proembryo derived from a zygote microinjected with an anti-CDC2 antibody 18 h after sperm–egg fusion (HAF) and cultured within ovule halves for 36 h following re-implantation. (**a**) Bright-field micrograph of the resulting multicellular structure. (**b**) Nuclei visualised by staining with the DNA-specific fluorescent dye SYTO Green. (**c**) Immunolocalisation of CDC2-associated immunoreactivity using the anti-CDC2 antibody. (**d**) Merged image of panels (**b**,**c**), illustrating the spatial relationship between nuclear DNA and CDC2-associated fluorescence signals. (**e**) Cell walls visualised by Calcofluor White staining. The atypical cellular organisation and division pattern indicate disturbed embryogenic development following interference with CDC2 activity. Scale bars = 50 µm.

**Figure 25:**
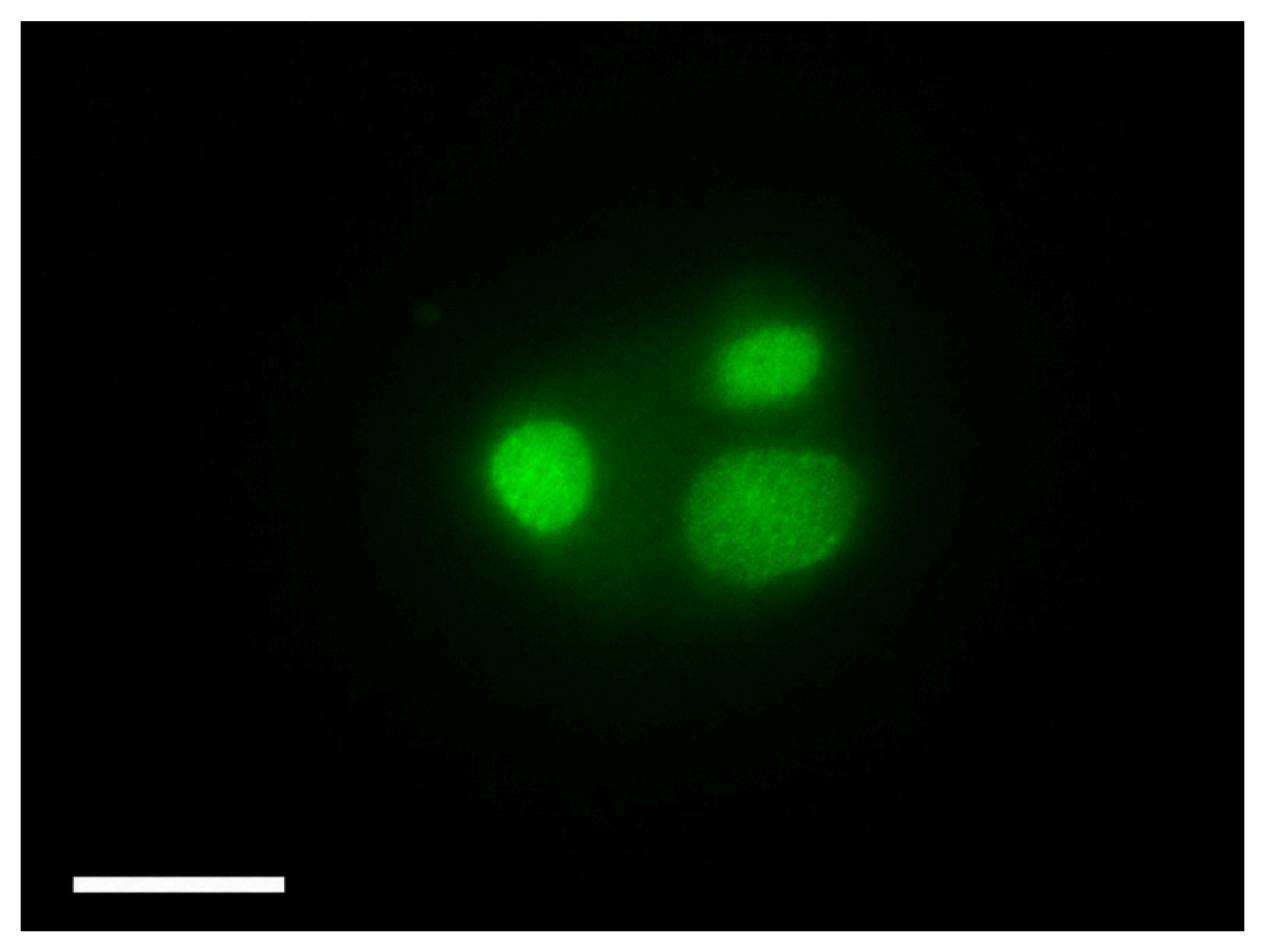
Divided zygote derived from an in vitro-fertilised wheat egg cell microinjected with an anti-CDC2 antibody 18 h after sperm–egg fusion (HAF). Nuclei were visualised by staining with the DNA-specific fluorescent dye SYTO Green, revealing the presence of daughter nuclei following cell division. Despite successful cytokinesis, the resulting cellular organisation differed from that typically observed during normal embryonic development. Scale bar = 30 µm.

By contrast, injections performed at later developmental stages produced substantially weaker effects. The timing of sensitivity indicates a restricted developmental interval during which interference with CDC2-associated processes strongly affects subsequent embryogenesis. Similar abnormalities were observed following injection of anti-PSTAIRE antibody (Figure 24 and Figure 25), consistent with findings in Fucus zygotes, where early interference with PSTAIRE-containing CDKs inhibits cell division and polarised development [61].

The use of anti-CDC2 and anti-PSTAIRE antibodies provided complementary immunological evidence concerning proteins recognised by these reagents. However, antibody recognition or localisation does not establish cyclin binding, the activation state of a kinase complex or its catalytic activity. Together with the pharmacological and microinjection results, these observations are consistent with an association between CDC2-associated processes and early embryonic development, but do not demonstrate that an active CDC2–cyclin complex was present or that CDC2 directly controls embryonic polarity.

### 2.9. Genistein Reveals a Functional Relationship Between Phosphorylation-Dependent Signalling and Polarity Establishment

**Figure 26:**
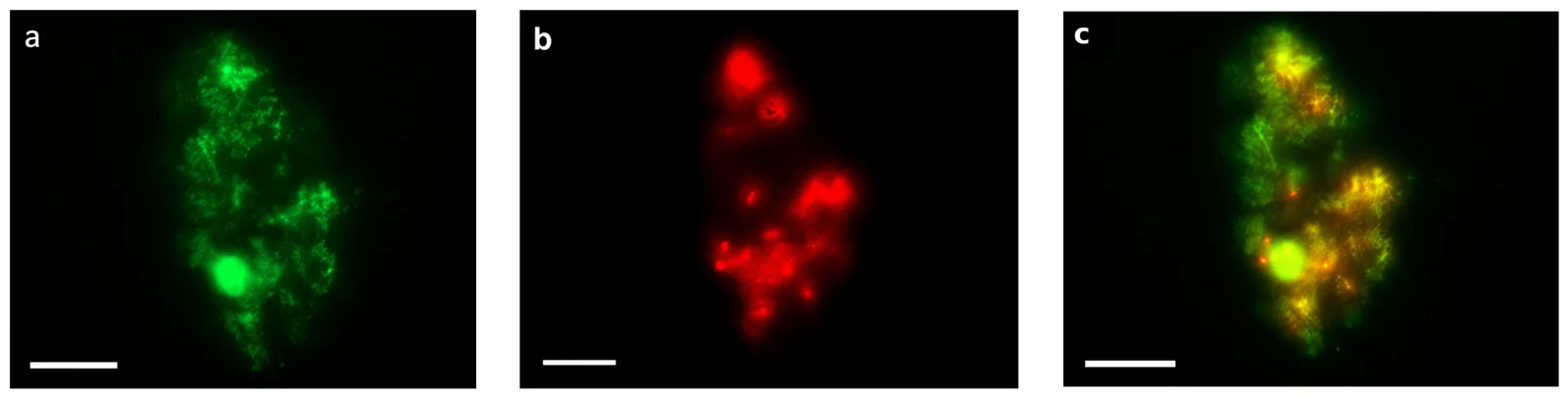
Typical effect of genistein treatment on tubulin organisation and p34cdc2 localisation in an in vitro-fertilised wheat egg cell. Genistein was applied 2 h following sperm–egg fusion (HAF). (**a**) Immunolocalisation of α-tubulin (green fluorescence), showing the organisation of the microtubule cytoskeleton. (**b**) Immunolocalisation of p34cdc2 (red fluorescence) using an anti-CDC2 antibody. (**c**) Merged image showing both fluorescence signals from panels (**a**,**b**), illustrating the spatial relationship between microtubule organisation and p34cdc2 localisation. Genistein treatment was associated with altered distributions of both signals and abnormal cellular organisation. Scale bars = 20 µm.

In order to investigate whether phosphorylation-dependent signalling pathways participate in the establishment of embryonic polarity, fertilised egg cells were exposed to the protein tyrosine kinase inhibitor genistein. The application of genistein resulted in developmental abnormalities that differed from the arrested phenotypes produced by CDK inhibition. Although cell division failure occurred in some treated zygotes, a number of them continued development and gave rise to multicellular structures exhibiting severe disturbances in morphology and cellular organisation (Figure 26).

The illustrated genistein-treated cell failed to display a clearly defined apical–basal axis; the frequency of this morphological pattern was not established. Treated cells displayed irregular organisation and lacked the characteristic polarity observed in untreated controls, although cell proliferation continued in a number of cases. Because genistein has multiple molecular targets, these abnormalities cannot be attributed specifically to inhibition of protein tyrosine kinases. Rather, the observations are consistent with disruption of phosphorylation-sensitive processes involved in polarity establishment, while other actions of genistein may also have contributed to the phenotype.

## 3. Discussion

The transition from a fertilised egg cell to a patterned embryo is one of the most fundamental developmental events in the life cycle of higher plants. In the course of this process, cell-cycle progression, cellular differentiation and polarity establishment represent an intertwining and complex set of molecular and cellular events which must be precisely coordinated to establish the body plan of the future sporophyte. Although the molecular mechanisms controlling cell-cycle progression have been extensively studied in plants, considerably less attention has been given to how these mechanisms interact with the developmental processes responsible for establishing embryonic polarity. By combining in vitro fertilisation, micromanipulation techniques, semi-in vivo zygote culture, immunolocalisation and pharmacological perturbation approaches, the present study provides evidence that CDC2-associated processes participate during this developmental transition and hints that cell-cycle regulation, cell polarity and cell differentiation are functionally interconnected during early wheat embryogenesis.

In this regard, the Fucus zygote provides a particularly informative comparative system. Fertilisation is external and large populations of synchronously developing zygotes can be obtained, allowing environmental cues that influence polarity to be manipulated experimentally [60]. By contrast, angiosperm female gametes and zygotes develop deeply embedded within maternal tissues, which greatly restricts direct experimental access. The first zygotic division in flowering plants nevertheless provides a particularly important developmental transition because it generates daughter cells with distinct developmental fates. The fertilised angiosperm egg cell therefore offers a valuable system in which to examine how cell-cycle progression is coordinated with polarity and cellular differentiation.

By combining in vitro fertilisation, micromanipulation and re-implantation of fertilised wheat egg cells, we obtained access to selected early developmental stages. Immunolocalisation of p34cdc2 and microtubules in different fixed specimens provided representative images that may inform future studies of developmental regulation and possible maternal influences; the present observations do not establish continuous protein dynamics or the frequency of individual patterns.

### 3.1. Re-Implantation into Maternal Tissues Is Compatible with Organised Embryo Development

One of the most significant observations emerging from the present work is the observation that some in vitro-fertilised egg cells can undergo organised embryonic development following re-implantation into maternal tissues. Following transfer into dissected ovules, the representative specimens included developmental stages resembling those observed during normal embryogenesis. These examples do not quantify the success rate of re-implantation or demonstrate a continuous developmental trajectory for an individual embryo [14,15,16,17,18,19,20,52,64,65].

The working hypothesis of our investigation was that maternal tissues may be required for maintaining or restoring the capacity of fertilised egg cells to subsequently stabilise a developmental axis. The observations are consistent with this possibility but do not directly test whether maternal tissues are necessary. The illustrated development after re-implantation indicates that embryogenic competence can be retained in some specimens and is compatible with a supportive role for the maternal environment; it does not establish which signals are involved or whether they are essential. Recent advances in embryology have increasingly highlighted the importance of maternal influences during the earliest stages of embryogenesis. Although much of the work related to plants has been performed in Arabidopsis, evidence from cereals indicates that maternal tissues contribute not only nutritional support but also positional information and developmental cues that influence embryo patterning. In cereals, where embryo development occurs within highly specialised maternal structures, interactions between the embryo and surrounding tissues may be particularly important. The present findings are therefore consistent with emerging views that embryogenesis should be considered not as an autonomous developmental programme but as a process occurring within a maternally regulated developmental environment.

The semi-in vivo system used in this study provides a unique experimental framework for investigating these interactions [62,63]. Unlike conventional in vitro culture systems, where isolated zygotes often suffered from aberrant development or unorganised callus formation, re-implantation preserves the intimate association between the developing embryo and surrounding ovular tissues while maintaining experimental accessibility. Consequently, this approach may prove valuable for future studies aimed at identifying maternal signals involved in polarity establishment and embryo pattern formation.

### 3.2. Stage-Associated p34cdc2 Localisation Patterns During Early Zygotic Development

The representative immunolocalisation images showed differences in p34cdc2 localisation at successive stages between fertilisation and the first zygotic division [18,19,20,35,36,37]. An important limitation of this analysis is that immunolocalisation required fixation; consequently, the temporal sequence was reconstructed from different zygotes collected at defined intervals rather than by time-lapse observation of the same cell. The observed differences are therefore compatible with stage-associated variation in p34cdc2 localisation but do not constitute direct evidence of intracellular translocation within an individual zygote. This distinction is particularly important when interpreting the apparent transition from nuclear accumulation to asymmetric nuclear distribution and subsequently to a predominantly cytoplasmic pattern. Their occurrence at developmental stages associated with zygotic polarisation suggests that CDC2-associated processes may be coordinated with developmental events preceding the first asymmetric division. The lack of reliable frequencies for the individual localisation patterns also prevents conclusions about their prevalence or obligatory succession.

Importantly, immunoblot analyses showed that p34cdc2 was detectable throughout the developmental stages examined. Consequently, the observed developmental regulation is unlikely to result from major changes in protein abundance. Instead, the data are compatible with differences in intracellular localisation, but do not establish the mechanisms responsible for those differences. Such regulation is consistent with what is known of cyclin-dependent kinases, whose activity is controlled through association with cyclins, phosphorylation-dependent activation and dynamic subcellular localisation. Although the findings presented here do not establish whether asymmetric localisation of p34cdc2 actively contributes to polarity establishment or merely reflects underlying developmental processes, they are consistent with spatial variation in p34cdc2 immunoreactivity at stages associated with acquisition of embryonic polarity. This observation provides an important conceptual link between cell-cycle regulation and developmental organisation [13,22,31,66,67,68,69,70,71,72].

### 3.3. Association of p34cdc2 with the Preprophase Band Links Cell-Cycle Regulation to Division-Plane Determination

A particularly noteworthy finding of the present study is the close association between p34cdc2 and the preprophase band. The PPB is a plant-specific microtubular structure that accumulates at the future division plane [36,73], hence representing one of the earliest manifestations of spatial information within a dividing cell. Therefore, the correct positioning of the PPB is pivotal for oriented cell division and ultimately for the proper morphogenetic architecture of tissues.

Based on the observation that p34cdc2 co-localised with the PPB [20,22,29,30,31,32,73], it may be assumed that CDC2-associated processes are closely associated with cellular structures that are responsible for the determination of the future division plane. Putative links between cyclin-dependent kinases and cytoskeletal organisation have also been reported in various eukaryotic systems, where these kinases assist in the coordination of morphogenesis with cell-cycle progression. Our assumption is further supported by the oryzalin-application experiments since the disruption of the PPB by oryzalin was accompanied by the loss of p34cdc2 localisation at the PPB. These findings indicate that localisation of the kinase within the future division plane depends upon the integrity of the microtubular cytoskeleton. Whether the PPB acts primarily as a localisation platform for CDC2-associated complexes or whether CDC2 activity contributes to PPB organisation remains unresolved. Nevertheless, the results are consistent with a spatial association between CDC2-associated processes and the cytoskeletal machinery responsible for division-plane determination. From a developmental perspective, this observation is particularly relevant because the first zygotic division establishes the apical and basal lineages of the embryo [18,19,20,22,31,32,35,36,37,73,74,75,76]. Any mechanism influencing division-plane specification during this stage has the potential to affect subsequent pattern formation. The association between p34cdc2 and the PPB therefore provides a plausible mechanistic connection between cell-cycle regulation and embryonic morphogenesis.

### 3.4. Functional Perturbation Experiments Reveal a Coupling Between Cell-Cycle Progression and Embryonic Pattern Formation

Compelling evidence deriving from the perturbation experiments implemented in our study supports a developmental role for CDC2-associated processes. Pharmacological inhibition of cyclin-dependent kinase activity, antibody-mediated interference with CDC2 function and inhibition of phosphorylation-dependent signalling all resulted in profound developmental abnormalities.

CDK inhibitors such as olomoucine, roscovitine and amino-purvalanol are purine analogues that act as competitive inhibitors for the ATP-binding site of kinases, effectively blocking CDK activity. The inhibitory effects of these compounds support a role for CDK-sensitive processes in normal progression through the first embryogenic cell cycle [69,77,78]. The effects of these treatments were not limited to inhibition of cell division. Rather than simply arresting development, many embryos exhibited marked abnormalities in their overall organisation. These observations indicate that interference with CDC2-associated processes does not necessarily prevent cell division altogether but instead affects the normal spatial organisation of the developing embryo. Amino-purvalanol-treated zygotes frequently developed two conspicuous intracellular domains separated by a distinct boundary-like region. Unlike the pronounced morphological asymmetry that characterises normal angiosperm embryogenesis, these domains were remarkably similar in size and appearance. This phenotype suggests that inhibition of CDK activity affects not only progression through the cell cycle, but also developmental processes involved in the establishment or maintenance of embryonic polarity and the execution of asymmetric cell division.

Similar conclusions can be drawn from the microinjection experiments. Anti-CDC2 injection shortly after fertilisation inhibited division in 20 of 25 zygotes (80.0%); representative images of affected specimens also illustrate abnormal cellular organisation. These observations do not establish the frequency of each distinct morphological phenotype. Comparable, although generally less pronounced, abnormalities were also observed after anti-PSTAIRE antibody injection and, to some extent, following genistein treatment. Because the anti-PSTAIRE antibody recognises a conserved motif present in multiple CDKs, these findings further support the view that progression through the cell cycle is closely coordinated with the mechanisms that establish embryonic polarity during wheat zygotic embryogenesis.

If CDC2 function were restricted exclusively to controlling cell-cycle progression, inhibition would be expected to produce developmental arrest. Instead, the occurrence of aberrant embryonic organisation suggests that CDC2-associated processes participate in developmental processes responsible for establishing or maintaining embryonic polarity [13,22,31,43,71,72].

Importantly, the present data do not demonstrate that CDC2 itself functions as a primary polarity determinant. Rather, they support the more conservative interpretation that CDC2-associated processes may be involved in the coordination of polarity development with cell-cycle progression. Perturbation was associated with abnormal embryonic organisation, although these experiments do not identify the underlying mechanism. This interpretation is consistent with concepts developed in other biological systems, where cyclin-dependent kinases act not merely as regulators of proliferation but as integrators of developmental and morphogenetic information. Our results suggest that a similar principle may operate during early plant embryogenesis [13,22,31,32,40,43].

### 3.5. Phosphorylation-Dependent Regulatory Networks and the Stabilisation of the Embryonic Axis

The phenotypes induced by genistein provide additional insight into the regulatory processes operating during early embryogenesis. Unlike the pronounced developmental arrest typically caused by direct inhibition of CDK activity, genistein treatment often allowed development to continue, although the resulting embryos displayed severe defects in organisation and polarity. Similar effects of genistein on zygotic polarity and embryo patterning have been described in Fucus [79].

The effects of genistein should nevertheless be interpreted cautiously. Although genistein has been widely used as an inhibitor of protein tyrosine kinase activity, it is a pleiotropic compound with additional molecular targets, including DNA topoisomerase II, and is also well known for its phytoestrogenic activity in animal systems [80,81]. Consequently, the developmental abnormalities observed following genistein treatment cannot be attributed exclusively to inhibition of tyrosine phosphorylation. The present results are therefore consistent with an involvement of phosphorylation-sensitive processes in polarity establishment, but do not identify a specific tyrosine kinase pathway.

### 3.6. The Role of CDC2 in Early Wheat Embryogenesis

Our findings indicate that CDC2-associated processes are closely linked to progression of the first zygotic division in *Triticum aestivum*. Microinjection experiments revealed a temporal sensitivity window shortly after sperm–egg cell fusion, during which interference with CDC2 strongly inhibited subsequent developmental progression (Figure 22 and Figure 23). The markedly weaker effect of antibody injection at 20 HAF further indicates that this sensitivity is greatest during the earliest stages of zygotic development. Comparable observations in Fucus show that CDK-like proteins participate in regulation of both the first cell cycle and polarised development [60,61]. Taken together, these findings are consistent with particularly close coordination between CDK-associated cell-cycle regulation and developmental patterning during early zygotic development. However, the present experiments do not resolve the individual contributions or potential functional redundancy of specific CDK family members in the wheat zygote. The [Ca^2+^] cytoscillations previously observed in isolated wheat egg cells provide an additional indication that signalling events are initiated very early after fertilisation, although their relationship to CDK regulation remains to be determined [82].

### 3.7. A Working Model for the Coupling of Cell-Cycle Regulation and Polarity Establishment in Wheat Embryogenesis

The collective evidence obtained in the present study supports a model in which cell-cycle regulation, cytoskeletal organisation and developmental polarity are integrated components of a common regulatory framework governing early wheat embryogenesis [18,19,20,33,35,36,37,74,75,83]. Following fertilisation, the zygote undergoes a series of highly coordinated developmental events including polarity acquisition, redistribution of p34cdc2, organisation of the microtubular cytoskeleton and specification of the future division plane. Successful execution of these processes results in asymmetric division and normal embryo pattern formation. In contrast, pharmacological and antibody-based perturbations were associated with impaired cell division or abnormal embryonic organisation, consistent with disruption of coordinated developmental processes.

Our findings suggest that CDC2 does not act as a polarity determinant per se, but rather that CDC2-associated processes contribute to the stabilisation and execution of developmental polarity programmes. In this framework, cell-cycle progression and morphogenesis do not appear to be independent processes. Instead, they seem to operate as intertwining components of an intricate developmental system in which successful embryogenesis depends upon their precise coordination [13,22,31,62,66,67,68,70]. The present work therefore suggests that cyclin-dependent kinase pathways occupy an important position at the interface between cell proliferation and developmental pattern formation [13,22,31,32,43,71,72].

Further investigation of the molecular mechanisms linking these processes will contribute to a more comprehensive understanding of embryogenesis in cereals and other flowering plants by shedding light on how the cell-cycle machinery and cell differentiation act in unison to produce the competent zygotic embryo.

## 4. Materials and Methods

### 4.1. Plant Material and Growth Conditions

Plants of an Italian bread wheat (*Triticum aestivum* L.) genotype (“Grano Tenero”) were cultivated in a controlled-environment growth chamber under a 16 h light/8 h dark photoperiod at 15/17 °C day/night temperatures and approximately 70% relative humidity. Light intensity was maintained at approximately 350 μmol pm^−2^ s^−1^. Spikes containing immature florets were harvested at developmental stages appropriate for egg-cell isolation. Ovaries were dissected under sterile conditions and used immediately for gamete isolation.

### 4.2. Isolation of Egg Cells and Sperm Cells

Egg cells were isolated according to the procedure previously established for wheat gametes by Pónya et al. [84]. Briefly, ovaries were dissected under osmotic stabilisation conditions and individual egg cells were released by micromanipulation. Isolated cells displaying intact morphology and normal cytoplasmic organisation were selected for subsequent fertilisation experiments. Sperm cells were isolated from mature pollen grains by applying hypo-osmotic shock and placed immediately into fusion droplets.

### 4.3. In Vitro Fertilisation Procedure

Gamete fusion was performed via electrofusion of isolated wheat egg cell–sperm cell pairs within a microdroplet environment (see Video S1). The fusion medium consisted of 600 mOsmol kg^−1^ mannitol supplemented with 2 mM CaCl_2_ (pH 6.0), maintained under mineral oil on a glass coverslip to serve as a micro-electrofusion chamber. Individual egg cell–sperm cell pairs were aligned using an alternating-current (AC) field generated by a CF-150 Cell Fusion Instrument (BLS, Budapest, Hungary). Dielectrophoretic alignment was achieved at a frequency of 1 MHz and field strength of 75 V cm^−1^. Following successful alignment, direct-current (DC) fusion pulses were applied according to the manufacturer’s technical specifications to induce membrane destabilisation and subsequent gamete fusion. The fusion products were immediately transferred to a microdroplet for further incubation and subsequent experimental manipulation under sterile conditions.

### 4.4. Microinjection Procedure

The transfer and microinjection of cells were performed following the procedures previously described in Pónya et al. [84] (see Video S2). A micromotor-driven manipulator (DC3-K; Applied Scientific Instrumentation, Eugene, OR, USA) was mounted onto a Nikon Diaphot 300 inverted microscope, equipped with differential interference contrast (DIC) optics. Cell injection was facilitated by a pneumatic microinjector (MPPI-2; Applied Scientific Instrumentation, Eugene, OR, USA). Cells were microinjected with either the p34cdc2 antibody or the monoclonal anti-PSTAIRE antibody at precisely defined intervals following IVF (in vitro fertilisation). The final concentration of the injected antibodies was 5 mg/mL in an injection buffer consisting of 10 mM HEPES, 100 mM KCl, pH 7.2. Injection volumes were maintained at approximately 10 pL per cell, as estimated based on the pressure and duration settings of the microinjector. Negative control experiments involved the microinjection of in vitro-fertilised egg cells from the same batch with the same injection buffer lacking the specific antibodies. After injection, the microcapillary was carefully withdrawn. All manipulations were carried out under sterile conditions in a mannitol-based isolation medium (600 mOsm/kg) overlaid with mineral oil to minimise evaporation. The development of injected zygotes was followed by time-lapse microscopy and compared with that of untreated control embryos to evaluate the effects of CDC2 inhibition on early embryogenesis.

### 4.5. Semi-In Vivo Re-Implantation System

To re-establish contact between the developing zygote and the surrounding maternal tissues while maintaining experimental access, a semi-in vivo re-implantation system was elaborated and used. Individual wheat ovules were carefully excised from ovaries of manually pollinated florets. For controlled fertilisation, spikes were emasculated at the late uninucleate microspore stage and hand-pollinated with mature pollen collected from donor plants after the embryo sacs had reached maturity. Following fertilisation, the ovules were isolated and bisected by a transverse cut perpendicular to the micropylar–chalazal axis. Only the half containing the embryo sac and the developing zygote was used as nurse tissue. These ovule halves were placed on N6Z medium supplemented with 0.4 M glucose and maintained under these culture conditions throughout the experiment. This approach preserved the immediate maternal and gametophytic environment surrounding the developing zygote while allowing direct experimental manipulation and observation [62,63]. Glucose served both as the primary carbon source and as an osmoticum, providing culture conditions comparable to those previously reported to be suitable for in vitro wheat zygote culture based on nurse-tissue culture systems. To assess developmental competence following in vitro fertilisation, isolated zygotes were re-introduced into embryo-sac-containing ovule halves and cultured under semi-in vivo conditions (see Video S3). Development of re-implanted zygotes was monitored by light microscopy and compared with developmental stages described for naturally developing wheat embryos.

### 4.6. Production of an Affinity-Purified Anti-p34cdc2 Antibody

To generate an antibody specific to the wheat p34cdc2 protein, a synthetic peptide corresponding to the 17 C-terminal amino acids of the deduced sequence was synthesised [85]. This peptide was conjugated to keyhole limpet hemocyanin (KLH) via the m-maleimidobenzoyl-N-hydroxysuccinimide ester (MBS) cross-linker and utilised for rabbit immunisation according to established protocols. For affinity purification, the synthetic peptide was conjugated to bovine serum albumin (BSA) and subsequently immobilised onto cyanogen bromide (CNBr)-activated Sepharose resin (Pharmacia, Uppsala, Sweden). The rabbit antiserum was passed through the affinity column in triplicate to ensure maximum binding. Following extensive washing with 10 mM Tris-HCl (pH 7.2) to remove non-specific components, the bound antibodies were eluted using 100 mM glycine (pH 2.5). The eluate was immediately neutralised, dialysed against an appropriate buffer, and concentrated via Centricon ultrafiltration. All immunological procedures were carried out as described by Harlow and Lane.

### 4.7. Protein Extraction, SDS-PAGE, and Immunoblotting

Total protein was extracted from in vitro-fertilised wheat egg cells collected at defined intervals after fertilisation. Protein concentrations were determined using a Bradford protein assay. Protein samples were resolved by 12.5% SDS-polyacrylamide gel electrophoresis (SDS-PAGE). Following electrophoresis, proteins were either stained with Coomassie Brilliant Blue R-250 to verify total protein integrity or electrophoretically transferred to nitrocellulose membranes for immunoblot analysis. To minimise non-specific binding, membranes were blocked with 5% non-fat dried milk in Tris-buffered saline with 0.1% Tween-20 (TBST) for 1 h at room temperature. Membranes were then incubated with the affinity-purified anti-p34cdc2 primary antibody (diluted 1:1000 in TBST) overnight at 4 °C. After washing with TBST, membranes were incubated with an appropriate horseradish peroxidase (HRP)-conjugated secondary antibody (diluted 1:5000). Immunoreactive proteins were visualised using Bio-Rad Clarity™ Western ECL Substrate. Chemiluminescent signals were captured using a Bio-Rad ChemiDoc™ imaging system (Bio-Rad Laboratories, Hercules, CA, USA). Equal protein loading was verified by re-probing membranes or by analysing the total protein profile from the corresponding Coomassie-stained gels.

### 4.8. Immunofluorescence Labelling

For immunolocalisation studies, isolated egg cells, zygotes, and embryos were immobilised on poly-L-lysine-coated microscope slides and fixed in a freshly prepared fixative containing 4% (*w*/*v*) paraformaldehyde, 5 mM EGTA, and 50 mM potassium phosphate buffer (pH 7.0) for 45–60 min at room temperature. Following fixation, cells were washed three times with phosphate-buffered saline (PBS) and permeabilised with 0.1% (*v*/*v*) Triton X-100 in PBS for 10 min. To improve optical clarity and reduce background autofluorescence, samples were subjected to a clearing step using a graded series of methanol washes (e.g., 50%, 70%, and 100% methanol in PBS) followed by rehydration. Slides were then blocked with 3% (*w*/*v*) bovine serum albumin (BSA) in PBS for 30 min. Samples were incubated with primary antibodies diluted in PBS containing 1% (*w*/*v*) BSA. Microtubules were labelled using a mouse monoclonal anti-α-tubulin antibody (Sigma-Aldrich, St. Louis, MO, USA) at a 1:50 dilutiona 1:50 dilution. The localisation of p34cdc2 was determined using an affinity-purified rabbit anti-p34cdc2 antibody at a 1:80 dilution. After incubation at 4 °C overnight, the slides were washed extensively in PBS and incubated for 1 h at room temperature with the following secondary antibodies: biotinylated goat anti-rabbit IgG (for p34cdc2 detection) followed by streptavidin-conjugated fluorophores, and fluorescein-conjugated horse anti-mouse IgG (Vector Laboratories, Newark, CA, USA) for tubulin visualisation. Nuclear staining was performed using 1 µg/mL 4′, 6-diamidino-2-phenylindole (DAPI) or 1 µMSYTO™ Green (Molecular Probes, Eugene, OR, USA) for 10 min in the dark. Cell walls were visualised by staining with 0.01% (*w*/*v*) Calcofluor White for 5 min. Finally, the samples were mounted in an anti-fade mounting medium (Vectashield, Vector Laboratories, Newark, CA, USA) to preserve fluorescence signal integrity during microscopic observation. Control experiments were performed by omitting the primary antibodies or using pre-immune serum to confirm the specificity of the immunolabelling.

### 4.9. Fluorescence and Confocal Microscopy

Fluorescence imaging was performed using an Olympus BX50 research microscope (Olympus Corporation, Tokyo, Japan) equipped with a 100× oil-immersion objective and coupled to a high-resolution Olympus DP70 colour CCD camera (Olympus Corporation, Tokyo, Japan). Confocal laser scanning microscopy (CLSM) was performed using a Bio-Rad MRC-1024 system (Bio-Rad Laboratories, Hercules, CA, USA) mounted on an Olympus BX50F4 microscope (Olympus Corporation, Tokyo, Japan). Fluorophores were excited and detected at 365/420 nm (DAPI), 488/525 nm (fluorescein and SYTO™ Green), and 350/450 nm (Calcofluor White). Images were acquired and analysed with the manufacturer’s LaserSharp 2000 software (version 5.2; Bio-Rad Laboratories, Hercules, CA, USA). Brightness and contrast were adjusted only by linear, uniform transformations applied to the entire image.

### 4.10. Pharmacological Treatments, Microinjection, and Kinase Inhibition

To examine the roles of cell-cycle regulation and spindle formation during early embryogenesis, zygotes were exposed to a range of pharmacological inhibitors. Stock solutions were prepared in dimethyl sulfoxide (DMSO) and diluted into the culture medium immediately before use; the final DMSO concentration never exceeded 0.1% (*v*/*v* [61]). Oryzalin (10 µM) was used to disrupt microtubule organisation, whereas olomoucine (100 µM), roscovitine (20 µM) and amino-purvalanol (10 µM) were applied to inhibit cyclin-dependent kinase (CDK) activity. Iso-olomoucine (400 µM) served as a negative control. To investigate the contribution of phosphorylation-sensitive processes to axis formation, zygotes were treated with genistein (50–100 µM). In the microinjection experiments, an anti-PSTAIRE monoclonal antibody was injected into the zygote cytoplasm to interfere with PSTAIRE-containing CDKs, following the approach used in Fucus zygotes. All treatments were applied at defined times after fertilisation, and solvent controls received the corresponding concentration of DMSO. After treatment, cells were fixed and processed for immunofluorescence as described in Section 4.8.

### 4.11. Experimental Design and Statistical Analysis

Developmental responses were assessed by monitoring cell division, polarity status and embryo morphology. Binary developmental outcomes are expressed as the number of responding zygotes relative to the total number examined [n/N (%)]. For quantitative analyses, N denotes the total number of cells examined within a given experimental group (100%), whereas n denotes the number of cells exhibiting the particular response or characteristic under investigation. The percentage of responding cells was calculated as (n/N) × 100. Experiments involving pharmacological treatments and microinjection were conducted over four independent experimental sessions using independently isolated and fertilised egg cells. Differences in categorical division outcomes between experimental groups were analysed using two-sided Fisher’s exact tests, with *p* < 0.05 considered statistically significant. Microscopy-based observations of cellular morphology and protein localisation were evaluated descriptively.

## 5. Conclusions

This study provides evidence that CDC2-associated processes are linked to cell-cycle progression and may be coordinated with polarity establishment and embryonic patterning during early wheat embryogenesis. The representative localisation patterns, PPB-associated immunoreactivity and developmental effects of pharmacological and antibody-based perturbations are consistent with an association between CDC2-associated processes, cytoskeletal organisation and division-plane specification. The present data do not establish the prevalence of the localisation patterns or a direct causal role for CDC2 in polarity establishment.

The semi-in vivo re-implantation system further indicates that the maternal ovular environment can be compatible with organised embryogenic development in some re-implanted specimens; stage-specific success frequencies were not established. Together, these findings support a model in which interference with CDC2-associated processes may affect cell-cycle progression and embryonic organisation, although the present experiments do not define a specific molecular mechanism linking these effects.

## Data Availability

Data supporting the findings of this study are available from the corresponding author upon reasonable request.

## Supplementary Materials

The following supporting information can be downloaded at https://www.mdpi.com/article/10.3390/ijms27219612/s1.
